# Scalable protein production by *Komagataella phaffii* enabled by ARS plasmids and carbon source-based selection

**DOI:** 10.1186/s12934-024-02368-3

**Published:** 2024-04-20

**Authors:** Florian Weiss, Guillermo Requena-Moreno, Carsten Pichler, Francisco Valero, Anton Glieder, Xavier Garcia-Ortega

**Affiliations:** 1 Christian Doppler Laboratory for Innovative Pichia pastoris host and vector systems, Institute of Molecular Biotechnology, Graz University of Technology, Graz, A-8010 Austria; 2 Christian Doppler Laboratory for Innovative Pichia Pastoris Host and Vector Systems, Department of Chemical, Biological and Environmental Engineering, Universitat Autònoma de Barcelona, Cerdanyola del Vallès, Bellaterra, 08193 Spain

**Keywords:** *Komagataella phaffii*, *Pichia pastoris*, Episomal ARS plasmid, Lipase *Ca*lB, Carbon source marker, *GUT1*, *TPI1*, Methanol-free bioprocesses

## Abstract

**Background:**

Most recombinant *Komagataella phaffii* (*Pichia pastoris*) strains for protein production are generated by genomic integration of expression cassettes. The clonal variability in gene copy numbers, integration loci and consequently product titers limit the aptitude for high throughput applications in drug discovery, enzyme engineering or most comparative analyses of genetic elements such as promoters or secretion signals. Circular episomal plasmids with an autonomously replicating sequence (ARS), an alternative which would alleviate some of these limitations, are inherently unstable in *K. phaffii*. Permanent selection pressure, mostly enabled by antibiotic resistance or auxotrophy markers, is crucial for plasmid maintenance and hardly scalable for production. The establishment and use of extrachromosomal ARS plasmids with key genes of the glycerol metabolism (glycerol kinase 1, *GUT1*, and triosephosphate isomerase 1, *TPI1*) as selection markers was investigated to obtain a system with high transformation rates that can be directly used for scalable production processes in lab scale bioreactors.

**Results:**

In micro-scale deep-well plate experiments, ARS plasmids employing the *Ashbya gossypii TEF1* (transcription elongation factor 1) promoter to regulate transcription of the marker gene were found to deliver high transformation efficiencies and the best performances with the reporter protein (*Ca*lB, lipase B of *Candida antarctica*) for both, the *GUT1-* and *TPI1-*based, marker systems. The *GUT1* marker-bearing strain surpassed the reference strain with integrated expression cassette by 46% upon re-evaluation in shake flask cultures regarding *Ca*lB production, while the *TPI1* system was slightly less productive compared to the control. In 5 L bioreactor methanol-free fed-batch cultivations, the episomal production system employing the *GUT1* marker led to 100% increased *Ca*lB activity in the culture supernatant compared to integration construct.

**Conclusions:**

For the first time, a scalable and methanol-independent expression system for recombinant protein production for *K. phaffii* using episomal expression vectors was demonstrated. Expression of the *GUT1* selection marker gene of the new ARS plasmids was refined by employing the *TEF1* promoter of *A. gossypii.* Additionally, the antibiotic-free marker toolbox for *K. phaffii* was expanded by the *TPI1* marker system, which proved to be similarly suited for the use in episomal plasmids as well as integrative expression constructs for the purpose of recombinant protein production.

**Supplementary Information:**

The online version contains supplementary material available at 10.1186/s12934-024-02368-3.

## Background

The methylotrophic yeast *Komagataella phaffii*, formerly known as *Pichia pastoris*, is a preferred microbial host for the production of recombinant proteins and metabolites in research and industrial applications [1]. Typical eukaryotic post-translational modifications enable the production of proteins which are otherwise difficult to synthetize or fold by more simple microbial hosts [2]. Most production strains are created by integration of the expression vector into the genome [3, 4], often using high DNA concentrations and increased selection pressure to support multi-copy integration. While this usually results in genetically stable clones at least for single copy strains, non-specific integration - of potentially multiple (often tandem) copies - might positively or adversely influence heterologous gene expression or protein secretion due to the affected genomic loci where the plasmid integrated via non-homologous end-joining (NHEJ) [5–7]. Multiple copies of similar expression cassettes integrated in close proximity are usually not genetically stable [8].

Certain applications like high-throughput library construction and screening or direct comparison of enzyme/promoter/secretion signal variants, however, would benefit from more efficient transformation and more uniform expression levels of different transformants by reduction of the widely described clonal variability [9, 10]. However, due to the low efficiency of homologous recombination in *K. phaffii*, so far transformation rates with linearized plasmids for site-directed integration were usually limited to max. 10^3^ – 10^4^ transformants per µg DNA - not enough for applications where high rates of transformation are desired [11]. The use of circular autonomously replicating episomal plasmids, on the other hand, has been shown to drastically increase transformation rates [12, 13]. In addition, the above-mentioned locus effects caused by integrative vectors might be alleviated [12]. In absence of a functional 2-micron plasmid for *K. phaffii*, such plasmids usually carry an autonomously replicating sequence (ARS) that serves as an origin of replication. Besides endogenous sequences like the well-studied *Pichia* ARS 1 and 2 (PARS1 and PARS2) and a mitochondrial ARS, sequences from *Candida boidinii* and *Kluyveromyces lactis* have been described and successfully implemented in *K. phaffii* so far [12–15]. But unlike the well-investigated 2-micron plasmids in *Saccharomyces cerevisiae* [16, 17], currently available ARS plasmid systems in *K. phaffii* were inherently unstable and require constant selection in order to be maintained [9, 10, 18]. After initial work with ARS-based plasmids by Cregg et al. [12]. those vectors were given up and integrative vectors were preferred due to higher stability of transformants and more reliable expression. About 20 years later the *HIS4* selection marker was replaced by the simpler and more selective Zeocin resistance marker [9] but also this was not leading to any breakthrough and no production processes employing episomal plasmid vectors were reported so far. Due to the inherent instability of all early-generation episomal plasmids, efforts were made to facilitate mitotic distribution by placing centromeric sequences on the plasmids [18, 19]. This seemingly stabilized the plasmids to approximately one copy per cell, but their large size and low transformation efficiencies compared to standard ARS plasmids still restrict their use for the desired applications in high throughput experiments. For all types of plasmids selection pressure was usually applied by the use of antibiotic or auxotrophy markers [9, 10, 14, 20]. While antibiotic resistance genes are convenient dominant markers in small-scale lab experiments, their use at large scale is not suited for most product applications and is often considered economically unfeasible [21]. Prototrophic markers, on the other hand, complement deficiencies of auxotrophic host strains and therefore provide advantages for the host cells on defined media [21]. This selection pressure, however, can be diminished by cross-feeding during the cultivation, potentially leading to marker-less subpopulations [22]. Alternatively, genes that are required for the utilization of certain carbon sources could be used as efficient and economically feasible selection markers as already demonstrated for *Schizosaccharomyces pombe* and *S. cerevisiae* over 20 years ago, reviewed by Kjeldsen et al. as well as patented by Kawasaki and Bell [23, 24]. Therefore, a similar strategy might be successful for *K. phaffii*.

For example, the *GUT1* gene, coding for a glycerol kinase essential for glycerol metabolization, has been used as a marker gene in a *gut1* deficient strain for the cultivation in defined media with glycerol as the sole carbon source by Näätsaari et al. [11], and was also implemented on ARS plasmids [13]. As a possible alternative *TPI1*, which codes for a triosephosphate isomerase that catalyzes the interconversion of dihydroxyacetone phosphate to glyceraldehyde 3-phosphate and thus essentially connects the glycerol metabolism to glycolysis, had been shown to serve as an auto-selection marker in *S. cerevisiae* [24], which means it even provides selective pressure in complex media [21]. For *K. phaffii* the *TPI1* marker had not been used so far. Both *GUT1* and *TPI1* require strains that are deficient in the respective genes, which are then complemented by the selection marker cassette present in the episomal plasmid.

The goals of this study were to evaluate and improve the *GUT1* marker system for its application in ARS plasmids and to be implemented - for the first time - in scalable bioreactor cultures. In addition, the idea was to compare this selection system to a possible new *TPI1* marker system in *K. phaffii*. For direct comparison, the production of *Candida antarctica* lipase B (*Ca*lB), which is of industrial interest for a wide range of applications, was used as a reporter protein for both systems. Combinations of 5 distinct promoters of different regulations driving the marker gene expression were tested together with the PARS1 sequence in terms of *Ca*lB reporter activity in *gut1* and *tpi1* deficient strains. The latter had to be generated specifically for this study, because - to the best of our knowledge - no studies regarding a deletion of *TPI1* in *K. phaffii* were published at the time of writing this publication.

## Results and discussion

### P_*AgTEF1*_ lead to the highest reporter activities in deep-well plate scale

In order to find a reliable episomal plasmid system that is based on selection pressure provided by the carbon source in the media, the native *TPI1* and a codon-optimized *GUT1* gene, both essential for glycerol metabolism, were tested in combination with 5 different promoters of various strengths on a plasmid harboring the PARS1 sequence and a *Ca*lB expression cassette as the reporter. All plasmids generated in this study are summarized in Table 1. After the transformation of the *tpi1* or *gut1* deficient strains (BSYBG11 Δ*tpi1* and BSY11G1) with the respective plasmids the transformation efficiencies were determined (Figure S1, Supplementary file 1) after 5 days of incubation at 28 °C. Most integrative plasmids ranged between 1.5 and 3 × 10^3^ CFU/µg with the exceptions of P_*ILV5*_ - *GUT1* where no transformants could be obtained and P_*GCW14*_ - *TPI1* which showed a transformation efficiency of 0.5 × 10^3^ CFU/µg. On the other hand, transformation efficiencies of *TPI1*-based episomal plasmids ranged from 1.0 to 1.4 × 10^6^ and *GUT1*-based episomal plasmids from 1.5 to 2 × 10^6^ CFU/µg DNA. Here, the P_*GCW14*_ - *GUT1* combination produced transformants of such small size that they were almost not visible or distinguishable from the background (Supplementary file 1, Figure S2), and therefore was incubated for one more day to obtain some colonies for inoculation. Thus, the determination of the transformation efficiency could not be done in a reliable way. These results support the information found in literature that ARS containing plasmids, given that the selection marker expression is properly adjusted, enable higher transformation rates in yeasts, or specifically *K. phaffii*, than integration vectors [12, 25].

**Table 1:** All plasmids generated in this study

Plasmid Number	Description	Selection marker	Name	Promoter	PARS1
1	Cloning vectors; not used in *K. phaffii*	*GUT1/Ampicillin*	pBSY3G	P_*GUT1*_	-
2			pBSY3G_PARS1	P_*GUT1*_	+
3			pBSY3G_Dα*Ca*lB	P_*GUT1*_	-
4			pBSY3G_PARS1_Dα*Ca*lB	P_*GUT1*_	+
5	*K. phaffii* expression vectors	*GUT1*	pBSY3G_Dα*Ca*lB_P_*AgTEF*1_	P_*AgTEF1*_	-
6			pBSY3G_PARS1_Dα*Ca*lB_P_*AgTEF1*_	P_*AgTEF1*_	+
7			pBSY3G_Dα*Ca*lB_P_*GCW14*_	P_*GCW14*_	-
8			pBSY3G_PARS1_Dα*Ca*lB_P_*GCW14*_	P_*GCW14*_	+
9			pBSY3G_Dα*Ca*lB_P_*GUT1*_	P_*GUT1*_	-
10			pBSY3G_PARS1_Dα*Ca*lB_P_*GUT1*_	P_*GUT1*_	+
11			pBSY3G_Dα*Ca*lB_P_*ILV5*_	P_*ILV5*_	-
12			pBSY3G_PARS1_Dα*Ca*lB_P_*ILV5*_	P_*ILV5*_	+
13			pBSY3G_Dα*Ca*lB_P_*TPI1*_	P_*TPI1*_	-
14			pBSY3G_PARS1_Dα*Ca*lB_P_*TPI1*_	P_*TPI1*_	+
15	*K. phaffii* expression vectors	*TPI1*	pBSY3T_Dα*Ca*lB_P_*AgTEF1*_	P_*AgTEF1*_	-
16			pBSY3T_PARS1_Dα*Ca*lB_P_*AgTEF1*_	P_*AgTEF1*_	+
17			pBSY3T_Dα*Ca*lB_P_*GCW14*_	P_*GCW14*_	-
18			pBSY3T_PARS1_Dα*Ca*lB_P_*GCW14*_	P_*GCW14*_	+
19			pBSY3T_Dα*Ca*lB_ P_*GUT1*_	P_*GUT1*_	-
20			pBSY3T_PARS1_Dα*Ca*lB_ P_*GUT1*_	P_*GUT1*_	+
21			pBSY3T_Dα*Ca*lB_P_*ILV5*_	P_*ILV5*_	-
22			pBSY3T_PARS1_Dα*Ca*lB_P_*ILV5*_	P_*ILV5*_	+
23			pBSY3T_Dα*Ca*lB_P_*TPI1*_	P_*TPI1*_	-
24			pBSY3T_PARS1_Dα*Ca*lB_P_*TPI1*_	P_*TPI1*_	+

**Fig. 1:**
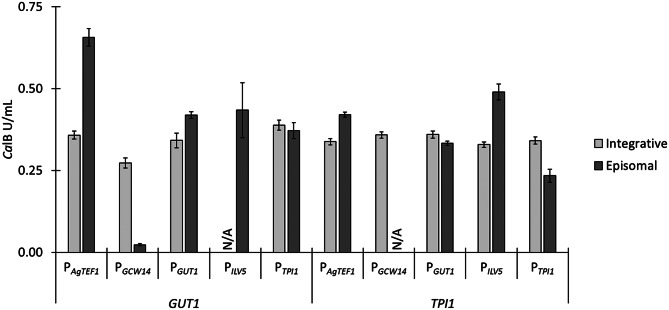
*Ca*lB reporter activities from transformants of episomal plasmid variants cultivated in micro-scale deep-well plates. Seven transformants of various ARS plasmids with different combinations of selection marker and its promoter, together with 7 colonies of the respective integrative references, were cultivated in 96 deep-well plates containing 250 µl BMG1. After 60 h of incubation at 28 °C and 320 rpm, 250 µl of BMG0.5 were added, followed by 50 µl BMG2.5 after 72 and 84 h to allow de-repression of P_*DC*_ and consequent reporter gene expression. Cultivations were harvested after 108 h of cultivation by centrifugation and *Ca*lB activities in the supernatants were evaluated

Seven transformants of each ARS plasmid (where applicable) were cultivated in 96 deep-well plates together with the integrative expression strain references (each reference strain was used to inoculate seven wells). The integrative reference strains are average production clones obtained from deep-well plate screening of 41 transformants of linear integrative plasmids (Figure S10 and S11, Supplementary file 1). The average measured *Ca*lB activities obtained from the deep-well plate screening are summarized in Fig. 1. The ARS plasmid carrying the P_*AgTEF1*_ - *GUT1* combination resulted in the highest reporter activity overall being about 83% higher than for the integrative reference. Plasmids with the promoters P_*GUT1*_ and P_*ILV5*_ driving *GUT1* expression lead to relatively similar volumetric activities, approximately in the range of the P_*GUT1*_ - *GUT1* integrative reference strain. The episomal P_*TPI1*_ and *GUT1* combination showed a slightly lower reporter activity, comparable to the respective integrative reference. Regarding the *TPI1* marker system, the plasmid with P_*ILV5*_ led to the highest *Ca*lB activities with about 49% surplus relative to the integrative reference. The ARS - P_*AgTEF1*_ - *TPI1* combination was the only other to surpass the integrative reference in the *TPI1* system (by 25%). Interestingly, the plasmids with P_*GCW14*_, which is one of the strongest constitutive promoters known in *K. phaffii* [26–28], barely show any *Ca*lB activity in the *GUT1* marker system and no activity at all in the *TPI1* system. While the P_*GCW14*_ – *GUT1* transformants reached comparable cell densities in deep-well plates despite their very small colony size on transformation plates, it was the opposite case for P_*GCW14*_ – *TPI1* transformants. Here, the transformants had a regular colony size but these cells showed basically no growth in deep-well plates. Since the expression of the gene *GCW14* has been shown to be very strong when *K. phaffii* is grown on glycerol and the activity of its promoter has been tested on various carbon sources, the regulation of P_*GCW14*_ should be suitable for the application tested in this study [26, 27, 29]. So, potentially, expression of the *GUT1* gene, driven by P_*GCW14*_ is too strong, perhaps even leading to the accumulation of Gut1p protein in the cell, thereby eliminating the vital necessity of the plasmid carrying the *GUT1* expression cassette. On the other side of the promoter strength spectrum, P_*ILV5*_ is said to show only about 15% of P_*GAP*_ strengh and is therefore the second weakest of the five promoters tested [30]. The *TEF1* promoter from *Ashbya gossypii* is assumed to show higher expression levels than the endogenous equivalent in *K. phaffii*, therefore ranging somewhere near the *GAP* promoter in terms of promoter strength [30, 31]. This strength was the most suitable for this plasmid and reporter setup among the tested promoters. The two native promoters of the tested selection markers, P_*GUT1*_ and P_*TPI1*_, were expected to lead to similar reporter activities as the respective integrative references when used with the respective reporter gene. But the episomal P_*GUT1*_ - *GUT1* plasmid surpassed the integrative reference in terms of *Ca*lB activity, while the episomal P_*TPI1*_ - *TPI1* plasmid could not maintain the expression levels achieved with the integrative control. P_*GUT1*_ is inducible by glycerol [32–34], but P_*TPI1*_ is generally considered to be constitutive, as shown in *Yarrowia lipolytica* [35], but similar work in *K. phaffii* indicates it is affected by the carbon source and shows only < 10% the strength of P_*GAP*_ when cultivatedin complex glycerol media [31].

The specific *Ca*lB activities (normalized to OD_600_) summarized in Figure S3 (Supplementary file 1) mostly indicate similar ratios between integrative control strains and respective episomal production clones (Fig. 1). For both marker systems, however, the episomal plasmids with P_*ILV5*_ driving marker gene expression seem to lead to higher biomass accumulation compared to the others, as the specific activity is reduced in relation to the other plasmid variants.

This information described above suggests that a certain level of promoter strength is key for maintaining the episomal plasmids without slowing down metabolism or reducing the selection pressure when trying to produce recombinant protein. The requirements might change with other recombinant proteins of interest as they exert different levels of stress on the host cell and therefore require other gene/transcript dosages for optimal production [36–38].

Interestingly, the standard deviations obtained when screening 7 separate transformants of ARS plasmids were generally low (< 9%, except for *GUT1*-P_*GCW14*_ and *GUT1*-P_*ILV5*_ plasmids), as they show values similar to the genomically stable integrative reference strains (standard deviations < 8%). This indicates their suitability for applications where clonal variability is not desired.

### Shake flasks cultivations

**Fig. 2:**
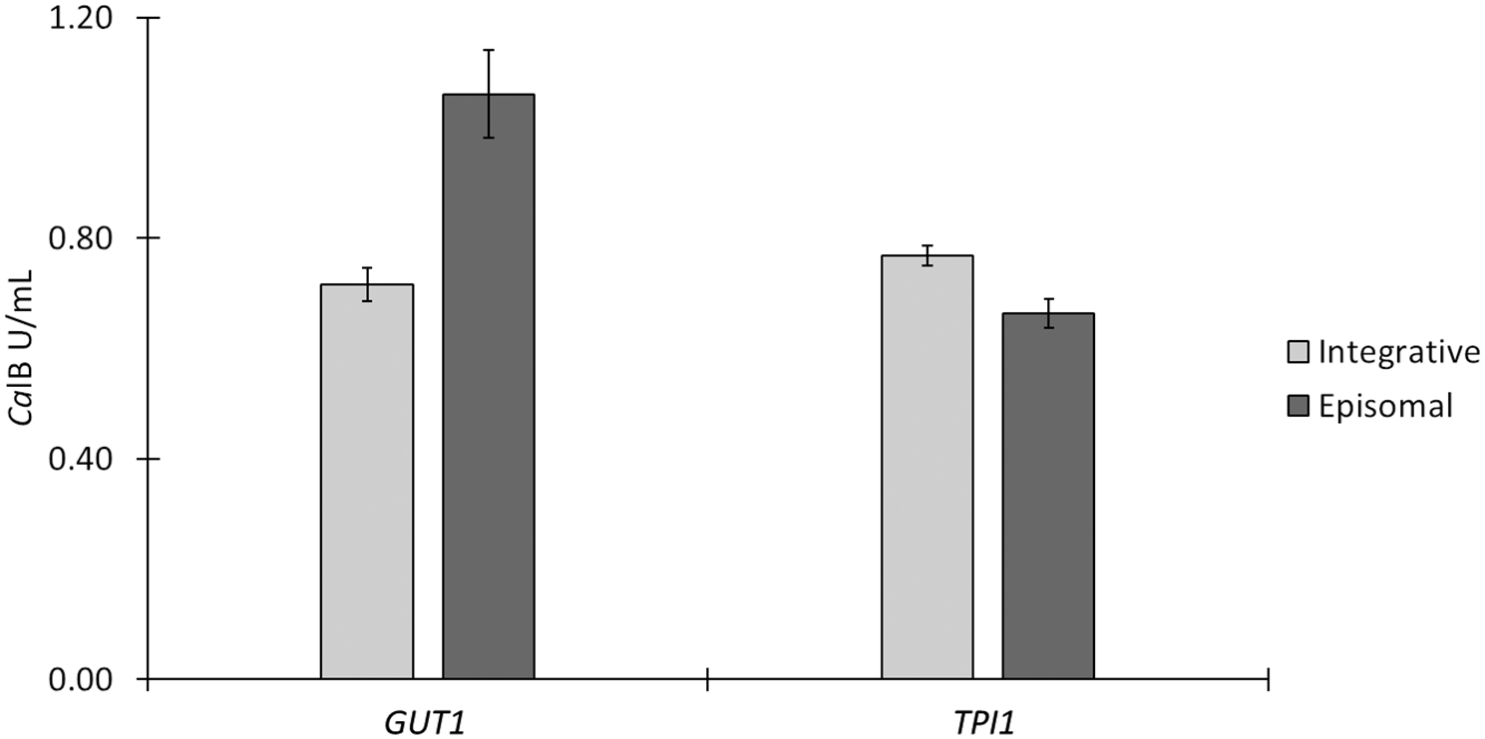
*Ca*lB reporter activities from 250 mL shake flask cultivations. Five transformants of ARS plasmids with P_*AgTEF1*_ driving the *GUT1* or *TPI1* marker expression were used to directly inoculate baffled 250 mL shake flasks containing 50 mL of BMG1. The respective average, integrative controls were cultivated in triplicates. After 60 h, 72 h and 84 h *GUT1* plasmid cultivations and after 100 h, 112 h and 124 h *TPI1* plasmid cultivations were fed with 500 µL of 50% glycerol. After 108 h the *GUT1* plasmid cultivations and after 148 h the *TPI1* plasmid cultivations were harvested and *Ca*lB activities were measured in the supernatants

One variant of both marker systems was scaled to 250 ml shake flask cultivations. For the *GUT1* marker system, the combination with P_*AgTEF1*_ was used for this experiment, as it has been shown to be the most promising in the initial micro scale screening. Even though P_*ILV5*_ resulted in slightly higher volumetric *Ca*lB activities with the *TPI1* system in deep-well plates, the plasmid with P_*AgTEF1*_ was preferred, because it showed more reliable specific activities (Figure S3, Supplementary file 1) and it can be assumed that the heterologous sequence of P_*AgTEF1*_ would reduce the potential risk of homologous recombination of the marker cassette with the genome compared to the endogenous P_*ILV5*_ sequence. Five fresh transformants of each episomal plasmid system were cultivated in separate flasks and each integrative reference strain was cultivated in triplicates in a fed-batch manner as described in the Materials and Methods section. The results are summarized in Fig. 2.

When employing the *GUT1* episomal system in 250 mL shake flasks with 50 mL media, *Ca*lB activities surpassed the average control strain with integrated expression cassette by 46% on average. Even though the relative surplus was not as big as in the micro-scale cultivation, the absolute volumetric activities were notably higher. The relative *Ca*lB activities coming from the episomal *TPI1* system in deep-well plates could not be reproduced in shake flasks as the integrative control led to 14% higher activities compared to the episomal clones. However, similar to the *GUT1* system, the absolute volumetric activities surpassed the deep-well plate cultivation. It was noteworthy that apparently, both episomal systems fell slightly short of expectations, as the surpluses to the respective references were smaller or even negative. The specific activity levels (normalized to the OD_600_) are summarized in Figure S4 (Supplementary file 1) show a similar pattern as the volumetric activity (Fig. 2), suggesting that differences in biomass are not causing the slight underperformance of the episomal systems. A possible explanation for this outcome could be that only glycerol without any YNB or phosphate buffer was used for feeding, as opposed to BMG media in the deep-well plate experiments, which might have led to a deficit of certain nutrients or an adverse change of pH. Another possibility could be that the productive phase was shorter in the larger shake flask volume compared to the deep-well plate, since also here single colonies were used for direct inoculation and therefore needed longer to deplete the initial glycerol before the P_*DC*_ promoter is derepressed [32, 39]. To ensure complete depletion of the initial carbon source (estimated based on final OD_600_ of the batch phase), the batch phase of the shake flask cultures of *TPI1* episomal transformants and the integrative reference strain were extended from 60 to 100 h. Still, the obtained *Ca*lB activities did not come close to the ones obtained from the *GUT1* system. Plasmid stability and copy numbers have not been assessed for shake flask cultivation, because effects of the cultivation method might have an impact on plasmid stability and copy numbers. Therefore, these values were only assessed for samples of the bioreactor cultivations described later in this study (Table S2, Supplementary file 1), as cultivation parameters can be controlled more strictly in bioreactors. Due to the combined reasons of longer cultivation times and nonetheless lower reporter levels, the *GUT1* based plasmid system was favored for fed-batch bioreactor cultivation tests in this study. While functional and applicable in principle, the *TPI1* marker system at least still would need more work for optimization. It could be worthwhile to consider altering the codon usage of the *TPI1* gene or testing other stronger, weaker or differently regulated promoters or testing destabilized protein variants of Tpi1p. As the regulation of the P_*AgTEF1*_ promoter seems suitable for both marker systems, testing of variants thereof could be also interesting.

Similar to the previously described low standard deviation for deep-well plate cultivations, the standard deviations of 5 single episomal transformants with P_*AgTEF1*_ driving marker expression in shake flasks, were also low, ranging from 2% (*TPI1* system) to 8% (*GUT1* system) respectively. Compared to the standard deviations of 3 cultures of each of the two genomically stable integrative reference strains (1% for *TPI1* and 4% for *GUT1* system) this suggests a high uniformity and low clonal variation in reporter activities among individual transformants of the episomal plasmids.

### Bioreactor cultivations

#### The physiological effect of the *GUT1* - deletion on growth capacity

Since the *K. phaffii* platform strain for the *GUT1* carbon source selection marker system has the native *GUT1* gene deleted, the capability of *GUT1* complemented transformants to grow on this substrate was initially characterized in comparison to the unmodified host. The physiologic effect on the producer strains was assessed through three independent glycerol-based batches for each expression system carried out in a 5 L fermenter under controlled conditions, where the yeast was growing at its maximum specific growth rate (*µ*_max_), which can be considered as a key parameter to evaluate and understand the growth performance and fitness of the strains [40].

The present study compared 3 different producer strains including: (1) the *gut1* - deletion strain in which plasmid 5 (Table 1) was integrated into the genome (integrative reference strain); (2) the *gut1* - deletion strain, which had been transformed with episomal plasmid 6 (Table 1), and (3) a control strain in which the native *GUT1* remains unmodified. Like the others, this control strain is based on the BSYBG11 platform and contains a single integrated *Ca*lB expression cassette regulated by the P_*DC*_ promoter but the native *GUT1* locus remains unaffected. By evaluating these three cases, the aim was to provide a comprehensive understanding of how this modification might affect the *µ*_max_ and subsequently the capacity of the cells to grow on glycerol as sole carbon source.

As described in previous works the *µ*_max_ during the batch phase can be accurately quantified based on the carbon dioxide evolution rate (CER) on-line data [41]. The *µ*_max_ of the strain with the native *GUT1* gene is 0.187 h^− 1^, which is slightly higher than the strain in which the *GUT1* plasmid number 5 was integrated (0.179 h^− 1^) and that of the strain containing the recombinant episomal *GUT1* plasmid number 6 (0.178 h^− 1^). The *µ*_max_ was robust along the whole batch cultures. The plots obtained regarding these determinations are included in the Supplementary file 1, Figure S5.

However, since the difference of these results was only in the 5% range, it can be assumed that the capacity to metabolize and grow on glycerol as the sole carbon source has not been significantly affected in the complemented strains. There was no significant difference between integrative and episomal complementation regarding growth on glycerol as sole carbon source and the high fitness of complemented strains was considered to be key for the success of this selection system for scalable production clones.

### Comparative performance of the expression strains in fed-batch cultures

Two biological replicates of fed-batch cultures were performed for each expression system to assess the performance of the episomal *K. phaffii* expression system in bioreactors, and to compare it with the average integrative expression system. The obtained data were also used to determine the key production rates and yields, as well as to assess the plasmid stability of the episomal system. To obtain comparative results, C-limited cultures at a constant specific growth rate of 0.05 h^− 1^ in which a pseudo-steady state is achieved was selected as a fed-batch strategy. It was based on the implementation of a pre-programmed exponential feeding profile that controls and maintains a constant *µ* by limiting the amount of C fed [42]. The final biomass of around 80 g L^− 1^ of DCW obtained after 30 h of feeding was very similar in both episomal and integrative expression systems (Table 2).

**Table 2:** Averaged values of key process parameters obtained by fed-batch cultivations. Nominal and experimental specific growth rate (*µ*), maximum specific growth rate (*µ*_max_), final product titer, final dry cell weight (DCW), substrate consumption rate (*q*_*S*_), specific *Ca*lB production rate (*q*_*P*_), biomass-to-substrate yield (*Y*_*X/S*_), product-to-biomass yield (*Y*_*P/X*_) and product-to-substrate yield (*Y*_*P/S*_). Results represent the average of two biological replicates. ± indicate SD

	Integrative	Episomal	Increase
Nominal *µ* (h^− 1^)	0.05	0.05	-
Experimental *µ* (h^− 1^)	0.0466 ± 0.0028	0.0472 ± 0.0028	+ 1.4%
Final product titer (AU · L^− 1^)	4756 ± 64	9906 ± 463	+ 108.3%
Final DCW (g L^− 1^)	77.18 ± 1.16	79.70 ± 1.24	+ 3.3%
*q*_*S*_ *(g*_*S*_ *· g*_*X*_^*−1*^ *· h* ^*− 1*^ *)*	0.0898 ± 0.0067	0.0893 ± 0.0008	- 0.56%
*q*_*P*_ (AU g_X_^−1^ h^− 1^)	3.36 ± 0.33	7.13 ± 0.54	+ 112.4%
*Y*_*X/S*_ *(g*_*X*_ *· g*_*S*_^*−1*^ *)*	0.519 ± 0.012	0.529 ± 0.027	+ 1.9%
*Y*_*P/X*_*(*AU *· g*_*X*_^*−1*^*)*	72.04 ± 5.73	150.87 ± 7.07	+ 109.4%
*Y*_*P/S*_*(*AU *· g*_*S*_^*−1*^*)*	45.96 ± 3.17	91.59 ± 3.11	+ 99.3

The *µ* achieved experimentally were in the range of the desired set-point (0.05 h^− 1^), confirming the successful implementation of the feeding profile and the fed batch strategy. In this sense, there were no significant disparities observed in the specific rates of substrate utilization (*q*_*S*_) or in the biomass-to-substrate yield (*Y*_*X/S*_). This confirmed, similar to the previously discussed results of the batch phase, that the growing capacity was not significantly affected by complementation of the *gut1* deficiency using the *GUT1* selection marker either in integrative or episomal plasmids.

The *Ca*lB production rates and yields of the reporter protein were also determined and compared between the alternative expression systems. Since the *CalB* expression is regulated by the de-repressible *P*_*DC*_ promoter which is repressed during the batch phase (C-excess conditions) and activated during the carbon limited fed-batch phase, the levels at the end of the batch phase were consistently low in comparison to the final titers. However, after de-repression significant differences in reporter enzyme activity were observed. The episomal system presented a two-fold higher enzymatic activity compared to the average integrative system at the end of the bioprocess. These increases can be seen in Table 2 in terms of titers, specific production rates (*q*_*P*_) and yields (both *Y*_*P/X*_ and *Y*_*P/S*_). Additionally, SDS page analysis was performed to verify that *Ca*lB activities correlate with protein levels in the supernatant (Figure S6, Supplementary file 1).

**Fig. 3:**
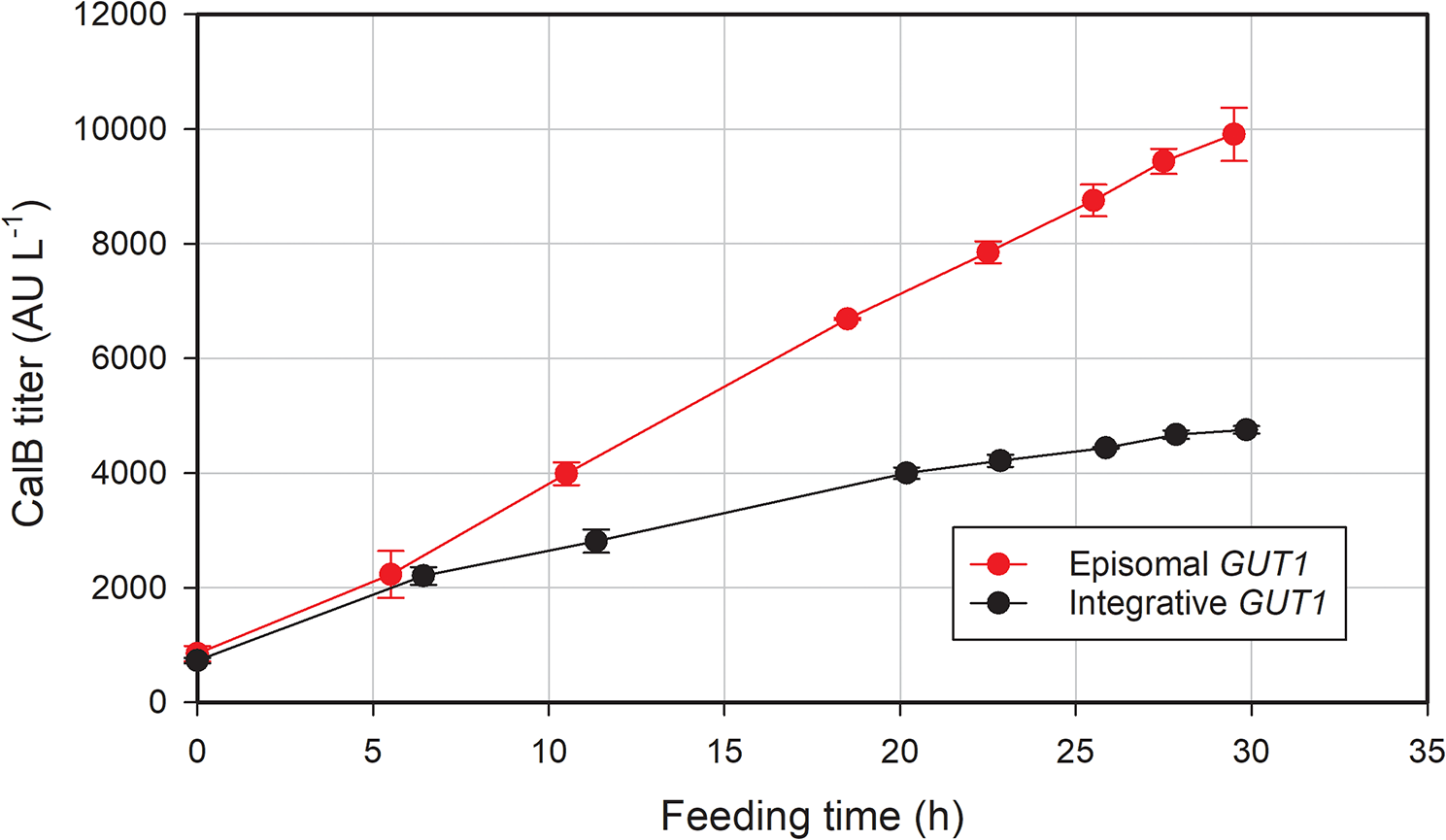
Time evolution of *Ca*lB titers during the fed-batch phase of the cultures. Results plotted are the average of two biological replicates

Interestingly, a consistent and continuous production of *Ca*lB along the culture for both the integrative and the episomal expression systems was observed (Fig. 3). These constant production profiles are usual for the widely studied genome-integrated systems but were surprising for the episomal system, based on the assumption that the employed ARS plasmids still will get lost to some extend over time. The data of this study indicated that the extrachromosomal plasmids were at least partially maintained in the cells throughout the whole cultivation and production process. For the first time - the stability of an episomal plasmid-based expression strain employing the *GUT1* carbon-source selection system in fed-batch bioreactor cultures was demonstrated to be sufficient to deliver significant amounts of recombinant product from scalable cultivation processes.

### Evaluating the episomal stability of *GUT1* - selected ARS plasmids

Several experiments were performed to investigate the stability of the ARS plasmids with *GUT1* marker in multi day bioreactor cultivations and compared to the strain with integrated expression cassette. First, samples from different cultivation and induction phases (E0 after inoculation, E1 at the end of the batch phase, E4 18.5 h after start of the fed-batch and E8 at the end of the cultivation) in bioreactors were diluted and plated on YPD and selective BMG1 agar plates to detect possible plasmid loss. Second, a comparative expression study was performed with deep-well plate cultivations and *Ca*lB activity measurements since episomal clones showed different characteristics compare to strains with integrated reporter cassettes. And third, analyses on DNA level had been performed by Southern blot analysis and quantitative PCR.

In the plating studies the number of colonies was determined after 5 days of incubation at room temperature. Cells that still carried the ARS plasmid were able to grow on both media, whereas cells without plasmid only grew on complex, non-selective YPD media. The ratio of cells still carrying the plasmid was calculated by dividing the colony count from selective agar plates (BMG1) by the total number of colonies grown on YPD plates. Interestingly, two types of colony sizes, normal and small, were observed on BMG1 plates after plating samples of the episomal system. After inoculation of liquid media only large colonies grew, indicating that the small colonies had been cells which had already lost the plasmid and perhaps grew to small colony size due to the availability of cellular storage compounds, not relying on external carbon sources for the initial cell divisions or impurities of the agar plates enabled such limited growth. Therefore, only large colonies were used for the calculations. In general, a decrease of plasmid-carrying cells (E) over time was observed compared to the integrative construct (I). In sample E0 from the start of the batch phase after inoculation, approximately 22% of the cells carried the plasmid. This number decreased to 12% in sample E1 taken at the start of the Fed-batch, indicating that about 50% of the plasmid containing cells lost the plasmid during the batch where expression of the reporter started. The lowest ratio of plasmid carrying cells was determined to be 7% in E4, showing a reduction of ~ 2/3 compared to the time of inoculation. In sample E8, however, the number of plasmid-carrying cells increased to 9% again. This indicated that during the carbon limited fed-bach cultivation, considering the increase of biomass, the fraction of plasmid containing, and therefore potentially productive cells, remained rather constant or even improved. These data were in line with the activity measurements where the productivity remained constant and did not drop as expected during longer cultivation and induction phases. The samples of the strain with the integrated cassette (I0-I8) did not change over time. These results are summarized in Table S2 (Supplementary file 1). While these findings indicated that the fraction of plasmid containing cells in the inoculum was already low and also during the batch phase when sufficient carbon source was present, the selection pressure was seemingly low whereas plasmid retention was relatively stable under carbon source limited conditions. Therefore, an improved procedure for the preparation of the inoculum might lead to further process improvements. The non-lethal character of this marker system needs to be considered: Cells without the plasmid do not die, as they would in antibiotic marker applications. They survive for a considerable time while not being able to grow or divide. Their metabolism is reduced and their participation in substrate consumption is assumed to be low, while their reporter production is necessarily zero without plasmid. If these stationary cells are provided with utilizable substrate, like YPD in this experiment, many of them would start growing again and form colonies, which made the subsequently calculated ratio of plasmid carrying cells look small. The high productivity of a cell population with a low fraction of cells carrying the expression cassette might be explained by higher copy numbers/cell for the episomal vectors compared to strains with integrated cassettes or also a better access for transcription at the episomal vector compared to the packed chromosomal expression.

**Fig. 4:**
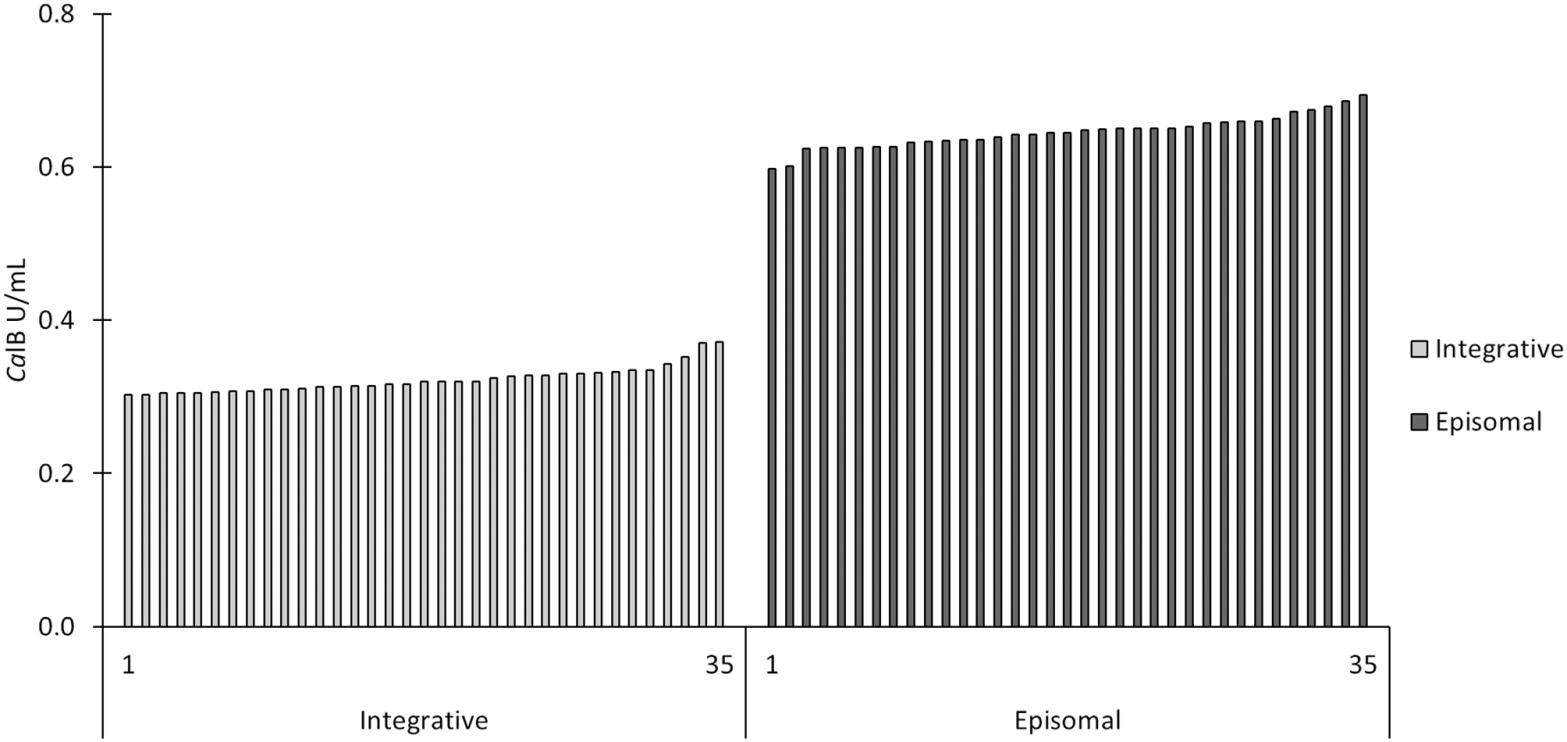
Re-screening of individual cells from bioreactor cultivation. Sample 8 from the bioreactor cultivations were plated on BMG1 agar plates to obtain single colonies. A 96 deep-well plate containing 250 µl BMG1 medium was inoculated with one single colony per well. After 60 h of incubation at 28 °C and 320 rpm, 250 µl of BMG0.5 were added, followed by 50 µl BMG2.5 after 72 h and 84 h to allow de-repression of P_*DC*_ and consequent reporter gene expression. Cultivations were harvested after 108 h of cultivation by centrifugation and *Ca*lB activities in the supernatants were evaluated. Each bar represents a single well of the deep-well plate

For the second experimental series, 35 single colonies of the plated samples I8 and E8 were inoculated in deep-well plates and cultivated following the same cultivation protocol as already described and reporter enzyme activities in the culture supernatant were determined. Activities in culture supernatants of episomal constructs were typically higher than those measured for strains with integrated cassettes and standard deviations indicating clonal variations or assay variations were low. Interestingly, no measurements for the episomal strains indicated a potential integration by changing expression characteristics to the typical behaviour of an integrative strain. If such integration event would occur in the late stage of the cultivation such modified phenotype would most likely not be seen by these assays but is also less relevant for the purpose of protein production within a typical cultivation and production period of 3–7 days. For the purpose of possible continuous production processes this would need a more detailed analysis. Therefore, the results summarized in Fig. 4 suggested that there was no significant or early integration event for the episomal plasmids. And, the enzyme titers based on activity measurements were twofold higher (201% ± 5%) compared to the *Ca*lB activity of integrative clones.

Finally, to further investigate the assumption that the *GUT1*-based ARS plasmids are extra-chromosomally stable and do not integrate into the genome on DNA level, also a Southern blot analysis employing a DIG-labeled *Ca*lB gene as a probe was performed. Genomic DNA was isolated from bioreactor samples E0, E1, E4 and E8, as well as from the integrative reference strain (3 separate preparations) and the empty parental platform strain *K. phaffii* BSY11G1. After detection, the blot (Figure S7, Supplementary file 1) showed bands at approximately 4 and 9.5 Kb that represent the ARS plasmid in the episomal bioreactor samples, which are not visible in the integrative strain or empty strain controls. These bands, however, seem to become weaker as the cultivation progresses, suggesting some plasmid loss in the cell population over time. This observation is consistent with the results obtained from the plating experiment described above as well as the copy number determination that is described in the following section. When looking at the integrative reference samples, faint but broad bands with sizes larger than 23 Kb, which corresponds to the expression cassette integrated into the genomic DNA fragments of large size caused by shearing forces during genomic DNA isolation, were visible. On the other hand, similar bands are missing in the episomal samples, indicating no or at least a low fraction of cells with integrated expression cassettes. This demonstrated that most of the observed reporter enzyme activity was generated by cells that contained non-integrated episomal expression plasmids.

Although the episomal *GUT1* ARS plasmid expression system is not entirely stable and a noticeable ratio of cells does not carry the plasmid in later phases of the bioreactor cultivation, the reporter activities obtained with the episomal system surprisingly still surpassed the integrative reference significantly. It was known from the literature that the currently available episomal plasmids do not segregate equally to the daughter cells in *K. phaffii* [9, 18], which could mean that, even though a certain proportion of cells might become plasmid-free, the other cells may carry multiple copies of the plasmid. Quantitative PCR was performed to analyze the change of total plasmid DNA in the cell population over time to validate the findings of the other performed analyses. The average episomal copy number (average of absolute and relative copies) did indeed decrease from approximately 3.6 ± 0.4 to 1.4 ± 0.2 from sample E0 to E4. Interestingly, in sample E8 the copy number increased again to 2.1 ± 0.4, again confirming the results from plating tests. Accordingly, since the fraction of cells containing plasmids was relatively low already after the batch cultivation this would suggest that cells of the episomal expression strains maintained significantly more than one plasmid copy per cell at the end of the bioreactor cultivation. Considering the average copy number determined is 2.1 per cell at the end of the cultivation and less than 10% of he cells in the population carried a plasmid, each of these cells would have to carry a high number of plasmids, calculated to be approximately 20 copies of plasmid. It was previously shown that *K. phaffii* can maintain high numbers of episomal plasmid copies in a study by Piva et al., where an ARS plasmid expressing eGFP and selected for with Zeocin was determined to be present in approximately 30 copies per cell on average [19].

## Conclusions

In this study the *GUT1* selection marker system for episomal ARS plasmids in *K. phaffii* was refined by employing the *TEF1* promoter of *A. gossypii* for the expression of the glycerol kinase 1 gene that allows growth on glycerol as the sole C-source. This plasmid system enabled recombinant production of lipase *Ca*lB on bioreactor scale that notably surpasses the average single copy integrative production clone by ~ 100%, and all performed analyses indicated that the plasmid was maintained as extrachromosomal vector by a significant fraction of the cell population throughout the cultivation process. For the first time scalable protein production by *K. phaffii* using episomal expression vectors was demonstrated. Additionally, the carbon marker toolbox allowing for efficient and antibiotic free selection was expanded with the *TPI1* marker with a *tpi1*-deficient *K. phaffii* platform strain. The high transformation rates and low clonal variations in reporter expression levels among transformants with these two episomal plasmid systems could be beneficial when screening mutagenesis libraries and when comparing the performance of key elements for recombinant protein production such as coding sequence, promoter, secretion signal, etc. Summarizing all advantages and disadvantages of both marker systems, the *GUT1* marker so far showed to be easier to use and therefore preferable. In addition to high transformation rates with episomal vectors the new plasmids enable simple and quick in vivo cloning in *K.phaffii* by simple co-transformation of linear gene constructs with vector backbones having overlapping regions of homology as described previously [10, 14]. Together with the new findings about scalable production this will facilitate the use of *K.phaffii* for a fast track from gene libraries to bioreactor scale processes for specific gene variants and products. *TPI1*, on the other hand, is described as an autoselection system in *S. cerevisiae*, applying selective pressure even in complex media [21, 24]. Therefore, by further improving and investigating the *TPI1* marker system with a codon optimized *TPI1*, different ARSs, other promotors of different strength and/or regulation, or destabilized or enzymatically less active Tpi1p variants the *TPI1* marker system might be improved in future and provide an attractive alternative to the *GUT1* based selection system. Such alternatives might be especially interesting for more demanding continuous cultivation processes.

## Materials and methods

### Chemicals

Restriction enzymes and Phusion HF DNA polymerase (used for standard PCR reactions according to the manual) were ordered from Thermo Fisher Scientific (Vienna, Austria) and single stranded DNA oligonucleotides were purchased from Integrated DNA Technologies (Leuven Belgium). Zeocin™ was obtained from InvivoGen (Toulouse, France), D(+)-biotin from Sigma-Aldrich (Vienna, Austria), Difco yeast nitrogen base w/o amino acids from Becton Dickinson (Schwechat, Austria) and Bacto yeast extract was obtained from (Thermo Fisher Scientific, Vienna, Austria). All other chemicals were purchased from Carl Roth (Karlsruhe, Germany).

### Plasmid construction

**Fig. 5:**
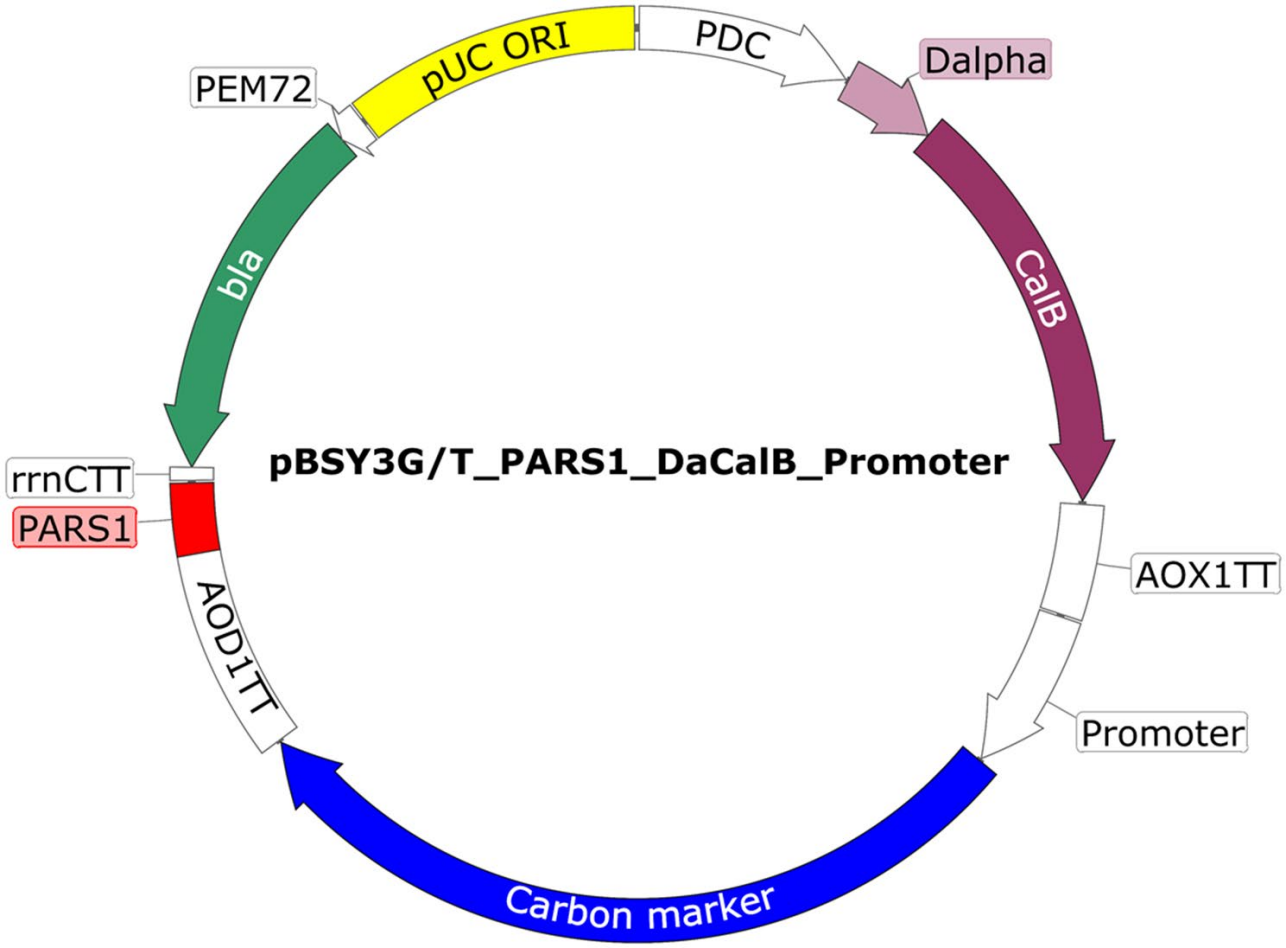
Vector map of a general ARS plasmid carrying a carbon marker cassette. Besides an origin of replication and an Ampicillin resistance cassette for cloning in *E. coli*, the episomal plasmids used in this study harbor the PARS1 sequence for the replication in *K. phaffii*, a reporter cassette with the de-repressable P_*DC*_ promoter driving *Ca*lB expression, whose secretion is facilitated by the D-alpha signal sequence, a deletion variant of the *S. cerevisiae* mating factor alpha signal peptide. Selective pressure in *K. phaffii* is provided by either *GUT1* or *TPI1* genes with one of the 5 tested promoters placed upstream

The plasmid pBSY3G, kindly provided by Bisy GmbH (Hofstaetten a. d. Raab, Austria), was linearized with *Xho*I and used for Gibson Assembly [43] with a PCR amplicon of the PARS1 sequence (Genbank: M11199.1; Primer 1 & 2), amplified from a CRISPR-Cas9 plasmid assembled by Weninger et al. [44], to form the plasmid pBSY3G_PARS1 [12]. All subcloning steps were performed in chemo-competent *Escherichia coli* XL1-blue cells. Correct plasmid assembly was verified by Sanger sequencing (Microsynth, Vienna, Austria) after each cloning step. These two plasmids were digested with *Sap*I to remove the stuffer fragment. The remaining vector backbones were used for Gibson Assembly with two PCR amplicons: the *Candida antarctica* lipase B gene (*CalB*) from Garrigós-Martínez et al. [28] (Primer 3 & 4) and the Dalpha leader, a modified sequence of the mating factor α signal peptide of *S. cerevisiae* taken from the commercially available plasmid pBSYA3S1G (Bisy GmbH) with primers 5 & 6. Then, the resulting pBSY3G_Dα*Ca*lB and pBSY3G_PARS1_Dα*Ca*lB were cut with *Nco*I and *Sac*I to remove the *GUT1* gene and promoter. The respective 4.1 Kb and 4.3 Kb, fragments were used for Gibson assembly with two inserts: (1) PCR amplicons of the codon-optimized *GUT1* gene from pBSY3G (Primers 7 and 8) or the native *TPI1* gene (Genbank protein ID: CAH2449166.1; Primers 9 and 10) and (2) PCR amplicons of promoters P_*AgTEF1*_ (from *Ashbya gossypii*) [45] or the endogenous P_*GCW14*_ [26], P_*GUT1*_, P_*ILV5*_ [30]or P_*TPI1*_ [31] via Gibson Assembly resulting in 20 plasmids total. The promoters were amplified from the respective genomes with primers 11 & 12/13, 14 & 15/16, 17 & 18/19, 20 & 21/22 and 23 & 24/25, respectively. This step was also performed with the P_*GUT1*_ to also introduce the novel restriction site between the promoter and marker gene. All primers used in this study are listed in Table S1 (Supplementary file 1). A general map of the ARS plasmids is shown in Fig. 5 and all created plasmids are summarized in Table 1.

### *K. phaffii* strain construction and transformation

The *K. phaffii* strain BSY11G1 (Δ*aox1*^*−*^*Δgut1*^*−*^), which is unable to grow on glycerol as sole carbon source and shows a Mut^S^ phenotype, was used as host for transformation of *GUT1* plasmids with glycerol-based selection. It was kindly provided by Bisy GmbH (Hofstaetten a. d. Raab, Austria) and was made by deletion of the *GUT1* gene of the killer plasmid-free Mut^S^ platform strain *K. phaffii* BG11 (Biogrammatics, Carlsbad, CA). This Δ*aox1* deletion strain was transferred to Europe and deposited as BSYBG11 strain by the Bisy GmbH.

For creation of a *tpi1* deficient strain, *K. phaffii* BSYBG11 (Bisy GmbH, Hofstaetten a. d. Raab, Austria) was transformed with 500 ng of *Smi*I linearized plasmid shown in Figure S8 (Supplementary file 1) in order to facilitate the homologous recombination-based knock out of *TPI1* as described by Cereghino et al. [46]. The transformation was plated on YPEtOH agar plates (2% (w/v) peptone, 1% (w/v) yeast extract, 0.1% (w/v) glucose, 2% (v/v) ethanol, 1.5 (w/v) % agar) containing 100 mg/L Zeocin and was incubated at 28 °C for 7–10 days [47, 48]. Very small colonies were picked and cultivated in 96 deep-well plates (Bel-Art Products, USA) containing YPEtOH media supplemented with 100 mg/L Zeocin for 4 days and then stamped on YPEtOH agar plates with Zeocin and BMG1 [BMG1: 1.34% (w/v) YNB, 1% (w/v) glycerol, 4 × 10^− 5^ % (w/v) biotin, 200 mM potassium phosphate buffer pH 6.0, 1.5% (w/v) agar] agar plates to screen for *TPI1* deficient clones. The knockout of *TPI1* was verified by colony PCR (Phire Plant Direct PCR Master Mix, Thermo Fisher Scientific, Vienna, Austria) (Primers 26 & 27) and subsequent Sanger sequencing (Microsynth, Vienna, Austria). A positive clone was then transformed with an ARS plasmid expressing a flippase gene (Figure S9, Supplementary file 1) for marker recycling. This transformation was performed as the previous one but was plated on YPEtOH agar plates containing 300 mg/L Geneticin. Successful marker recycling was again confirmed by genotyping and the strain was cleared from the ARS plasmid by growing it on non-selective YPEtOH plates. This *K. phaffii* BSYBG11 Δ*tpi1* strain was whole genome sequenced (Illumina, Macrogen, Seoul, South Korea) and is available upon request from BioGrammatics. The Illumina reads were mapped to the *K. phaffii* reference as provided by Sturmberger et al. (Genbank: GCA_900235035.2) and the data was analyzed according to Schusterbauer et al. [49]. The raw Illumina sequencing reads are available from the ENA database under project PRJEB73854 and relevant analysis results are summarized in the Supplementary file 2. The new *tpi1* deficient strain was used as transformation host with *TPI1* based vectors.

*K. phaffii* BSY11G1 and BSYBG11 Δ*tpi1* were transformed with 500 ng *Smi*I linearized plasmid variants without ARS sequence (pBSY3G_Dα*Ca*lB_X and pBSY3T_Dα*Ca*lB_X, respectively) for integration and 20 ng circular ARS plasmid variants (pBSY3G_PARS1_Dα*Ca*lB_X and pBSY3T_PARS1_Dα*Ca*lB_X, respectively) similar to the protocol described by Cereghino et al. [46]. The OD_600_ of the main cultures were 0.8 and 80 µl cells were used for transformations. 920 µl of buffered minimal media containing glycerol as sole carbon source (BMG1) were added to the transformations after electroporation and transformations were regenerated for 20 min at 28 °C and 600 rpm on a shaking platform. 20 µl of ARS plasmid transformations and 100 µl of integrative plasmid transformations were plated on BMG1 agar plates in triplicates for transformation efficiency determination and were incubated for 5 days at 28 °C.

41 transformants of each integrative plasmid were cultivated and screened as described in the following paragraphs to identify average neutral clones for *Cal*B production to be used as integrative reference for the respective episomal plasmid transformants. Figures S10 and S11 (Supplementary file 1) show the volumetric *Ca*lB activity landscapes of the transformants cultivated for the screenings. For each plasmid, clone 21 – the median – was picked and used for further experiments as the respective integrative control. It should be mentioned here, that no transformants of the plasmid 11 with P_*ILV5*_ driving *GUT1* expression could be obtained, even after re-sequencing the plasmid and multiple transformation attempts.

### Small scale cultivations and *Ca*lB enzyme assay

Freshly grown ARS plasmid transformants and integrative strains were cultivated in 96 deep-well plates (Bel-Art Products, USA) containing 250 µL BMG1 similar to Weis et al. [50] with some adaptions: After incubating the deep-well plates for 60 h at 28 °C and 320 rpm, the cultivations were fed with 250 µL of BMG0.5 [BMG containing 0.5% (w/v) glycerol], as well as 50 µL of BMG2.5 [BMG with 2.5% (w/v) Glycerol] after 72 h and 84 h of cultivation in order to allow de-repression of the carbon-source repressed P_*DC*_ promoter [32, 39], which was used for expression of the *CalB* reporter gene. The P_*DC*_ promoter is a 500 bp fragment of the *K. phaffii* catalase gene *CTA1* (sometimes also annotated as *CAT1*). The OD_600_ was measured and the supernatant was harvested after 108 h by centrifugation with 3000 g for 10 min.

For the first upscaling experiments, 250 mL baffled shake flasks containing 50 mL of BMG1 media were inoculated with freshly grown ARS plasmid transformants (1 colony per flask) or a single colony of the integrative reference strain and after 60 h, 72 h and 84 h 250 µL of 50% (w/v) Glycerol were added. The initial batch phase was elongated for another 40 h when cultivating transformants of the *TPI1* plasmid system due to slow growth rates and to ensure depletion of the initial glycerol supplemented in the media. The cultivations were harvested by centrifugation at 3000 g for 10 min.

Determination of the enzymatic *Ca*lB activities in the supernatants was performed as described by Krainer et al. [51], but the pH was set at 7 and the kinetics were measured for 3 min in 30 s intervals.

### Bioreactor cultivations

#### Inoculum preparation

The inoculate for bioreactor cultures were grown for approximately 24 h in 1 L baffled shake flasks at 25 ºC, 150 rpm, in BMG1 medium to reach an OD_600_ of 10. The shake flasks, each filled with 200 mL of culture medium, were initially inoculated with cryo-stocks of the selected producer clones. Subsequently, the cultures were allowed to grow, and cells were harvested via centrifugation, and then resuspended in the batch medium before being introduced into the bioreactor as the inoculum to obtain a starting OD_600_ of 1 in the bioreactor. When preparing the inoculum for cultivating the episomal production system, it was ensured to use a fresh single colony transformant to inoculate the shake flask.

### Fed-batch cultivations

The selected operational mode for the bioreactor cultures was a fed-batch. The cultures were performed in duplicates using a 5 L Biostat B fermenter (Sartorius Stedim, Goettingen, Germany), with an initial volume of 2 L. The batch medium contained per liter of destilled water: citric acid 1.8 g, glycerol 40 g, (NH_4_)_2_HPO_4_ 12.6 g, MgSO_4_·7H_2_O 0.5 g, KCl 0.9 g, CaCl_2_·2H_2_O 0.02 g, antifoam Glanapon 2000kz (Bussetti & Co., GmbH, Vienna, Austria) 0.05 ml, biotin 0.4 mg, and 4.6 ml PTM_1_ trace salts solution. The PTM_1_ solution contained per liter: CuSO_4_·5H_2_O 6.0 g, NaI 0.08 g, MnSO_4_·H_2_O 3.36 g, Na_2_MoO_4_·2H_2_O 0.2 g, H_3_BO_3_ 0.02 g, CoCl_2_ 0.82 g, ZnCl_2_ 20.0 g, FeSO_4_·7H_2_O 65.0 g, and 5.0 ml H_2_SO_4_ (95–98%). The feeding fed-batch medium contained per liter of distilled water: glycerol 400 g, MgSO_4_·7H_2_O 6.45 g, KCl 10 g, CaCl_2_·2H_2_O 0.35 g, antifoam Glanapon 2000kz 0.2 ml, biotin 0.2 mg, and 1.6 ml PTM_1_ trace salts solution. The specific growth rate (*µ*) during the fed-batch was fixed at 0.05 h^− 1^ by a preprogramming exponential feeding rate. During the whole process, Temperature was controlled at 25 °C and pH at 5.0 with NH_4_OH 15% v/v. Throughout the fermentation, aeration was maintained at 1 vvm. Stirring was varied (600–1200 rpm) in order to keep the dissolved oxygen always above 30%. The procedure is described in detail elsewhere [42].

### Biomass analyses

Biomass concentration, measured as dry cell weight (DCW), was determined following the method outlined in a previous work [52]. The Relative Standard Deviation (RSD) for this measurement was found to be below 5%.

### Quantification of the carbon source and byproducts

The quantification of glycerol, and potential fermentation by-products (ethanol, arabitol, and succinate) was determined using High-Performance Liquid Chromatography (HPLC). Details regarding the column and software utilized for this analysis can be found in the work by Garcia-Ortega et al. [53]. The obtained RSD for the HPLC-based analysis was below 1%.

### Off-gas analyses

The molar fraction of the off-gasses CO_2_ and O_2_ was monitored by a BlueInOne Cell gas analyzer (BlueSens, Herten, Germany) during fed-batch cultivations. The oxygen uptake rate (OUR), carbon dioxide evolution rate (CER), their specific rated (*q*_*O2*_ and *q*_*CO2*_), and respiratory quotient (RQ) were calculated taking the off-gas pressure and humidity measurement into account, as previously described [54]. RSD was less than 5% for all determinations.

### Key parameters determination

Specific rates and yields were calculated using on-line and off-line data. The calculation was based on the equations derived from material balances. The determination details were described in previous works [55].

In this study the following parameters were determined: the specific growth rate (*µ*), maximum specific growth rate (*µ*_max_), glycerol consumption specific rate (*q*_S_), *Candida antarctica* lipase B (*Ca*lB) protein specific production rate (*q*_P_), carbon dioxide emission rate (CER) and oxygen uptake rate (OUR).

### Enzymatic activity determination in supernatants of bioreactor cultures

The *Ca*lB production was quantified by the determination of its enzymatic activity in the sample supernatants. This determination was performed as described in previous work [56]. RSD was always below 5%.

### Plasmid copy-number determination

Plasmid copy numbers of the first episomal bioreactor cultivation were determined for bioreactor samples taken from the beginning of the batch phase (E0), at the end of the batch phase (E1), after 18.5 h into the fed-batch (E4) and at the end of the bioreactor cultivation (E8) of the ARS plasmid transformant by real-time PCR (qPCR) with the respective integrative reference strain as reference. The Luna® Universal qPCR Master Mix (New England Biolabs, Frankfurt am Main, Germany) was used according to the manufacturer’s manual in an Applied Biosystems™ 7500 Real-Time PCR System (Waltham, Massachusetts, USA). Genomic DNA was isolated according to Hoffman and Winston [57]. qPCR was performed with primers to amplify the reporter gene *Ca*lB (primers 28 & 29), as well as with primers for amplification of the single copy housekeeping gene *ARG4* (Argininosuccinate lyase gene; primers 30 & 31) as internal reference [58]. In addition to that the genome of the integrative reference strain was Illumina sequenced (Macrogen, Seoul, South Korea). Reads were mapped to the *K. phaffii* reference as provided by Sturmberger et al. (Genbank: GCA_900235035.2) including the integration vector sequence and bioinformatically analyzed to verify single copy integration via mean coverage as described by Schusterbauer et al. [49]. The raw Illumina sequencing reads are available from the ENA database under project PRJEB73854 and relevant analysis results are summarized in the Supplementary file 2.

### Southern blot

To detect potential integration events of the ARS plasmid, Southern blot was performed. 20 µg of the gDNA preparations was also used for qPCR and an additional preparation from the empty background strain *K. phaffii* BSY11G1, as well as 10 ng and 200 ng of isolated plasmid were loaded on an 0,75% agarose gel without ethidium bromide (80 V for 2 h). A second gel was prepared with Ethidium bromide and 0.5 µg of the gDNA samples as well as 10 ng and 200 ng of isolated plasmid were loaded on the gel. For detection of the reporter gene *CalB*, a probe was produced by PCR with primers 28 & 32 and the plasmid as a template and was DIG-labeled using the DIG DNA Labeling and Detection Kit (Roche, Mannheim, Germany) following the manufacturer’s manual. The blotting and hybridization steps were performed as described by Green and Sambrook [59, 60] in an aqueous hybridization buffer and with a DIG-labeled probe. The probe was detected using the DIG DNA Labeling and Detection Kit.

### Determination of plasmid stability

To calculate the ratio of cells in the bioreactor samples E0, E1, E4 and E8 that still contain the episomal ARS plasmid, approximately 0.2 × 10^− 7^ OD_600_ units were plated on YPD (2% peptone, 1% yeast extract, 2% glucose, 1.5% agar) and BMG1 agar plates. The colonies were counted after 5 days at 28 °C and the ratio was calculated by dividing the number of colonies on selective agar plates (BMG1) by the number of colonies on non-selective agar plates (YPD).

### Electronic supplementary material

Below is the link to the electronic supplementary material.

Supplementary Material 1

Supplementary Material 2

## Data Availability

The datasets and materials used and/or analyzed during the current study are available from the corresponding author on reasonable request. The raw Illumina sequencing reads are available from the ENA database under project number PRJEB73854.
