# Supplementary material for: Scalable protein production by Komagataella phaffii enabled by ARS plasmids and carbon source-based selection
Source: Microb Cell Fact. 2024 Apr 20;23:116. doi: 10.1186/s12934-024-02368-3 (PMC11031860; doi:10.1186/s12934-024-02368-3)
Supplement: Supplementary file 1 — Supplementary Material 1 [file 12934_2024_2368_MOESM1_ESM.pdf]

Scalable protein production by *Komagataella phaffii* enabled by ARS plasmids and carbon source-based selection

Florian Weiss[1], Guillermo Requena-Moreno[2], Carsten Pichler[1], Francisco Valero[2], Anton Glieder[1] *, Xavier Garcia-Ortega[2]

[1] Christian Doppler Laboratory for Innovative Pichia pastoris host and vector systems, Institute of Molecular Biotechnology, Graz University of Technology, A-8010 Graz, Austria

[2] Christian Doppler Laboratory for Innovative Pichia pastoris host and vector systems, c/o Department of Chemical, Biological and Environmental Engineering, Universitat Autònoma de Barcelona, 08193 Bellaterra (Cerdanyola del Vallès), Spain.

Corresponding author:

Phone: +43 (316) 873 - 4074

Fax: +43 316 873 9302

e-mail: a.glieder@tugraz.at

Table S1. List of primers used in this study.

Number	Name	Sequence 5' - 3'
Primer 1	PARS1_PstI_AODTT	GGATCTGATTACCTTAGCTGCAGTCGAGATAAGCTGGGGGAAC
Primer 2	PARS1+KpnI_rrnCTT	CCTTAGCGAAAGCTAAGGATTTTTTTTAGGTACCTCGACAATTAATATTTACTTATTTTG
Primer 3	AOX1TT start rev	GGCATTCTGACATCCTCTTG
Primer 4	CalB fwd	TTGCCTTCAGGTTCAGACCC
Primer 5	Dalpha_fwd_PDC	ACTTGCTCTAGTCAAGACTTACAATTAAAGAATTCATGAGATTCCCATCTATTTTCACCG
Primer 6	Dalpha_rev_CalB	GAAGGCTGGGTCTGAACCTGAAGGCAAAGCTTCGGCCTCTCTCTTC
Primer 7	GUT1 fwd	ATGGGAAAGGACTACACCCC
Primer 8	AODTT start rev	CTCTCTAAATAATCGTAAGGTGTCAATTCC
Primer 9	TPI fwd	ATGGTACGTGCATTGAGTCTCATAAG
Primer 10	TPI+NcoI_AODTT	CTCTAAATAATCGTAAGGTGTCAATTCCATGGCTAGTTTCTGGAATTAATAATGTCCACG
Primer 11	PAgTEF1+SacI_AOX1TT	GATTAAGTGAGACCTTCGTTTGTGCGAGCTCAGCTTGCCTCGTCCCCGC
Primer 12	PAgTEF1+BamHI_GUT1	CAAGAGGGGTGTAGTCCTTTCCCATGGATCCTTTTGTTTATGTTCGGATGTGATGTGAG
Primer 13	PAgTEF1+BamHI_TPI1	CTTATGAGACTCAATGCACGTACCATGGATCCTTTTGTTTATGTTCGGATGTGATGTGAG
Primer 14	PGCW14+SacI_AOX1TT	GATTAAGTGAGACCTTCGTTTGTGCGAGCTCGGGTGAAAGCCAACCATCTTTG
Primer 15	PGCW14+BamHI_GUT1	CAAGAGGGGTGTAGTCCTTTCCCATGGATCCTTTTGTTGTTGAGTGAAGCGAGTG
Primer 16	PGCW14+BamHI_TPI1	CACTTATGAGACTCAATGCACGTACCATGGATCCTTTTGTTGTTGAGTGAAGCGAGTG
Primer 17	PGUT1+SacI_AOX1TT	GATTAAGTGAGACCTTCGTTTGTGCGAGCTCATACCGAAAGGTAAACAACTTCGG
Primer 18	PGUT1+BamHI_GUT1	CAAGAGGGGTGTAGTCCTTTCCCATGGATCCTATAGTAGATATATCTGTGGTATAGTGTG
Primer 19	PGUT1+BamHI_TPI1	TTATGAGACTCAATGCACGTACCATGGATCCTATAGTAGATATATCTGTGGTATAGTGTG
Primer 20	PILV5+SacI_AOX1TT	GATTAAGTGAGACCTTCGTTTGTGCGAGCTCTTCAGTAATGTCTTGTTTCTTTTGTTGC
Primer 21	PILV5+BamHI_GUT1	AAGAGGGGTGTAGTCCTTTCCCATGGATCCAAATTTTTTCGGATAATTTTTTAAAGCGAG
Primer 22	PILV5+BamHI_TPI1	TATGAGACTCAATGCACGTACCATGGATCCAAATTTTTTCGGATAATTTTTTAAAGCGAG
Primer 23	PTPI1+Sac1_AOX1TT	GATTAAGTGAGACCTTCGTTTGTGCGAGCTCTTCAACGAGACACTCTTCCGTC
Primer 24	PTPI1+BamHI_GUT1	AAGAGGGGTGTAGTCCTTTCCCATGGATCCTGTGTTTGTGATAGATCTTGTATATCAATG
Primer 25	PTPI1+BamHI_TPI1	TATGAGACTCAATGCACGTACCATGGATCCTGTGTTTGTGATAGATCTTGTATATCAATG
Primer 26	TPI check fwd	CCCTCAAATGATGTCTCCTCTG
Primer 27	TPI check rev	GAAGGAGATAATACGAGGGGTG
Primer 28	CalB sense	CAGTTTTGGACGCAGGTTTGAC
Primer 29	CalB antisense	GTTCCAGTACCTGGGACAAGC
Primer 30	ARG4 sense	TCCTCCGGTGGCAGTTCTT
Primer 31	ARG4 antisense	TCCATTGACTCCCGTTTTGAG
Primer 32	CalB antisense 2	TGTGTCAATCCACCAGCGTTTC

Table S2. Plating experiment for the determination of plasmid carrying cells.

Integrative				Episomal			
Sample	Colony count		Cells with plasmid	Sample	Colony count		Cells with plasmid
	YPD	BMG			YPD	BMG	
I0	207 ± 8	202 ± 3	97%	E0	202 ± 14	45 ± 7	22%
I1	47 ± 5	45 ± 7	97%	E1	52 ± 4	6 ± 1	12%
I4	219 ± 11	212 ± 6	97%	E4	280 ± 30	20 ± 2	7%
I8	150 ± 13	147 ± 17	98%	E8	85 ± 1	8 ± 3	9%

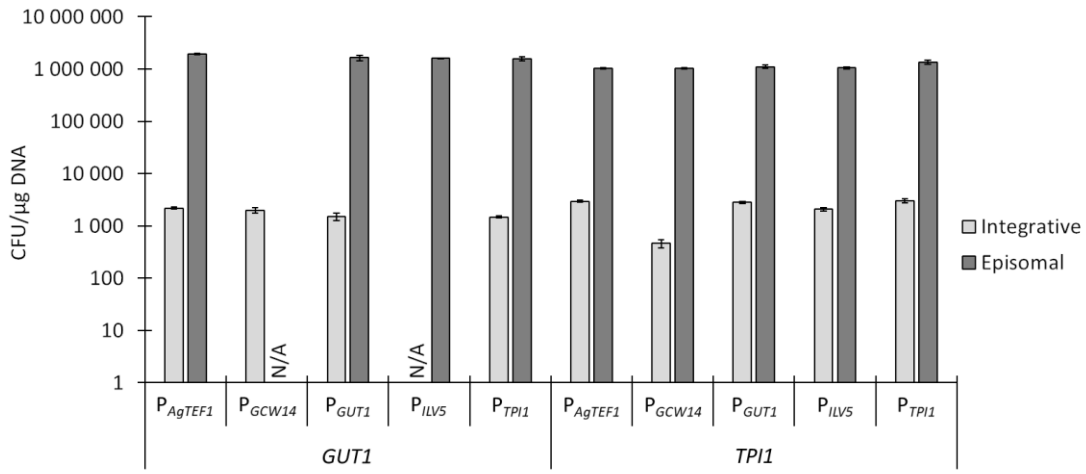

Figure S1. Transformation efficiencies. Integrative and episomal plasmids generated in this study were used to transform *K. phaffii* according to Lin-Cereghino *et al.* (2005). Transformations were plated on buffered minimal media plates containing 1 % glycerol as sole carbon source after 20 minutes of regeneration at 28 °C and 600 rpm agitation. Transformant selection was based on *TPI1* or *GUT1* marker systems. The obtained colonies per mL and µg of DNA were evaluated after 5 days of incubation at 28 °C.

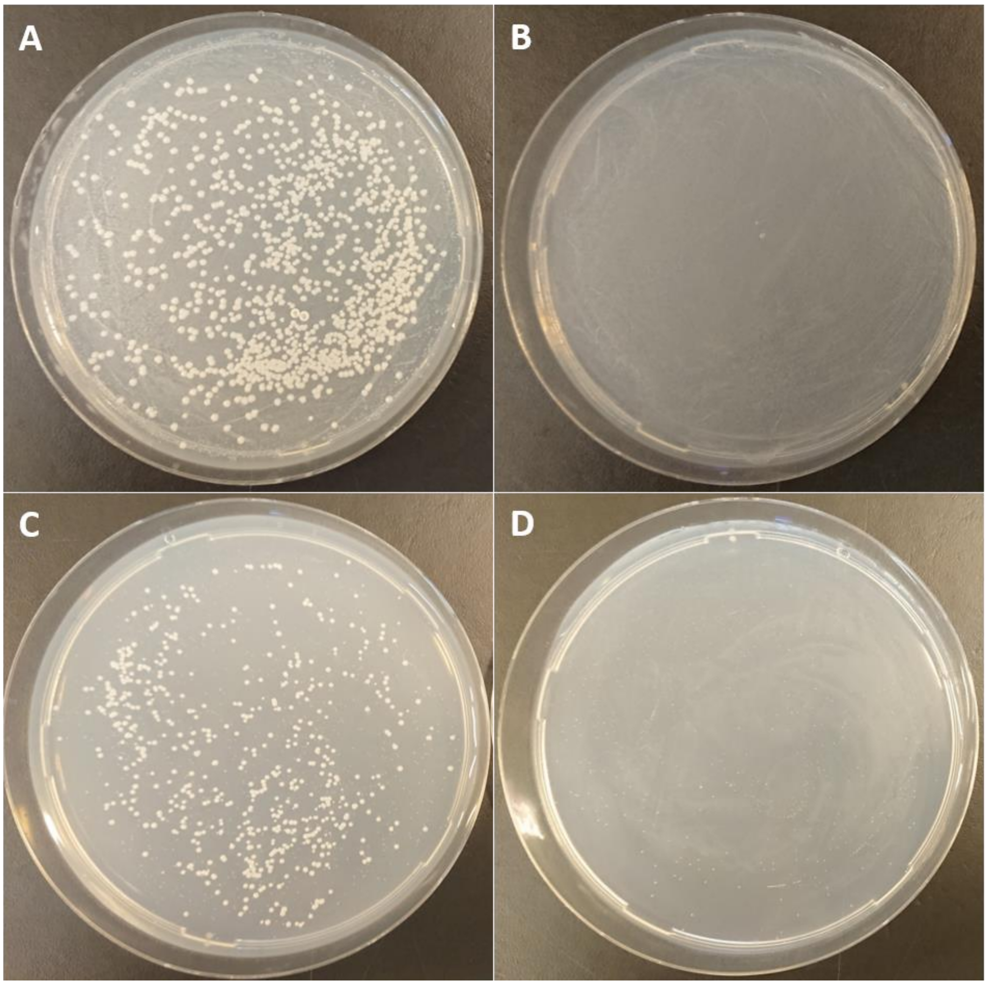

Figure S2: Pictures of transformation plates. Pictures of BMG1 transformation plates were taken after 5d incubation at 28 °C. *K. phaffii* strain BSY11G1 was transformed with plasmid #6 (Table 2) (A) or plasmid #8 (B) and strain BSYBG11 Δ*tpi1* was transformed with plasmid 16 (C) and without DNA (D) according to Lin-Cereghino *et al.* 2005. The transformations were regenerated in BMG1 medium for 20 minutes and plated on BMG1 agar plates for selection of transformants. Image A and C represent selection plates with transformants that are clearly distinguishable from the background (*GUT1*- or *TPI1*-deficient strain without plasmid). Image B shows a transformation plate with non-visible or non-distinguishable transformant colonies. Image D represents a negative control of *TPI1*-plasmid transformants with reduced background compared to *GUT1* transformation plates (small white dots are tiny air bubbles that occur during incubation).

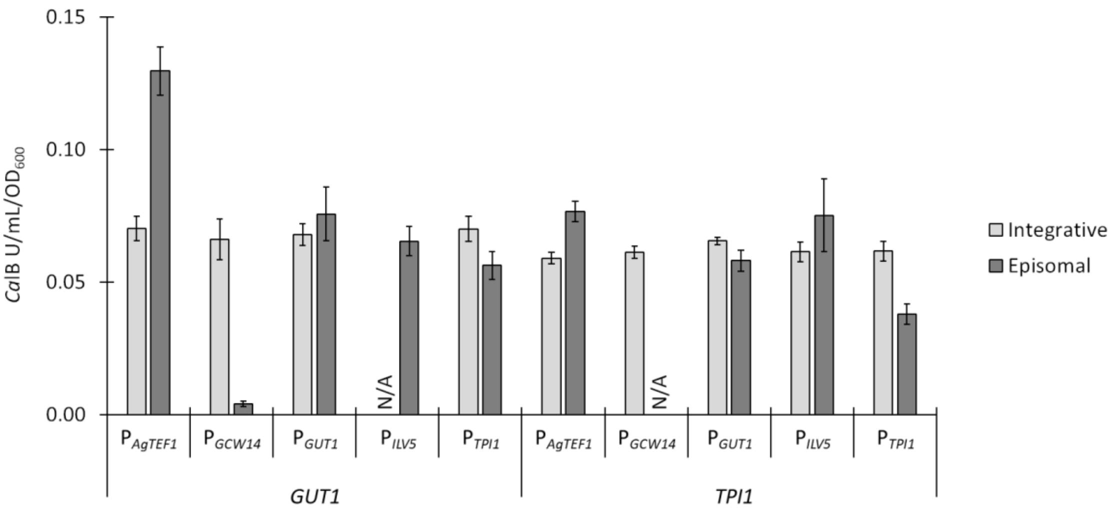

Figure S3: Specific *Cal*B activities obtained from deep-well plate cultivation of episomal plasmid transformants. Seven transformants of various ARS plasmids with different combinations of selection marker and its promoter, together with 7 colonies of the respective integrative references, were cultivated in 96 deep-well plates containing 250 µl BMG1. After 60 h of incubation at 28 °C and 320 rpm, 250 µl of BMG0.5 were added, followed by 50 µl BMG2.5 after 72 and 84 h to allow de-repression of P_{DC} and consequent reporter gene expression. Cultivations were harvested after 108 h of cultivation by centrifugation and *Cal*B activities in the supernatants were evaluated. The obtained *Cal*B activities were normalized to the OD_{600}, which was measured using 200 µl of culture in microtiter plates using a Synergy MX plate reader (Biotek, Winooski, VT, USA).

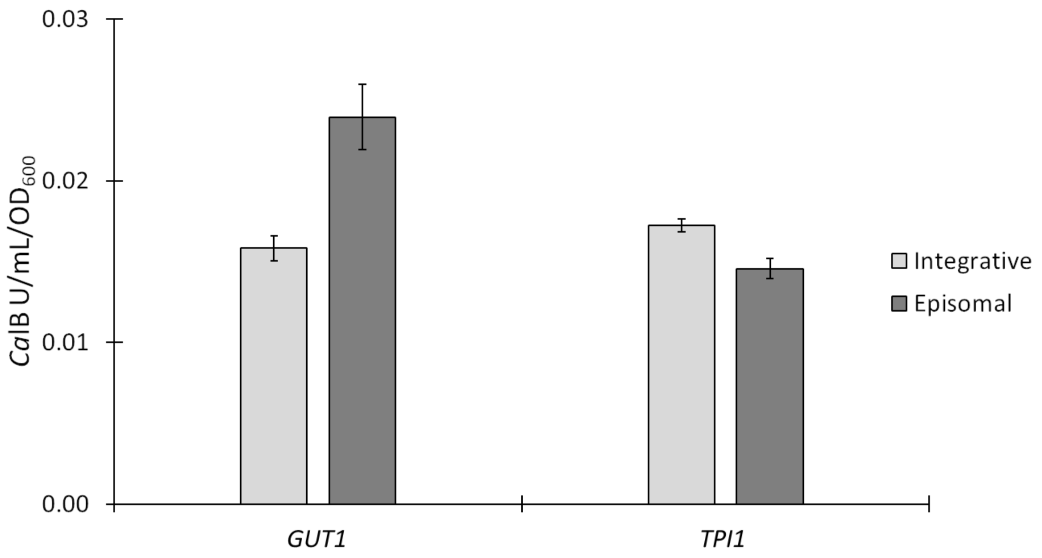

Figure S4: Specific *Cal*B activities obtained from 250 mL shake flask cultivations. Five transformants of ARS plasmids with P_{AgTEF1} driving the *GUT1* or *TPI1* marker expression were used to directly inoculate baffled 250 mL shake flasks containing 50 mL of BMG1. The respective average, integrative controls were cultivated in triplicates. After 60 h, 72 h and 84 h *GUT1* plasmid cultivations and after 100 h, 112 h and 124 h *TPI1* plasmid cultivations were fed with 500 µL of 50 % glycerol. After 108 h the *GUT1* plasmid cultivations and after 148 h the *TPI1* plasmid cultivations were harvested and *Cal*B activities were measured in the supernatants. The obtained *Cal*B activities were normalized to the OD_{600}, which was measured in half-micro cuvettes with 12.5 mm distance using an Eppendorf BioPhotometer plus.

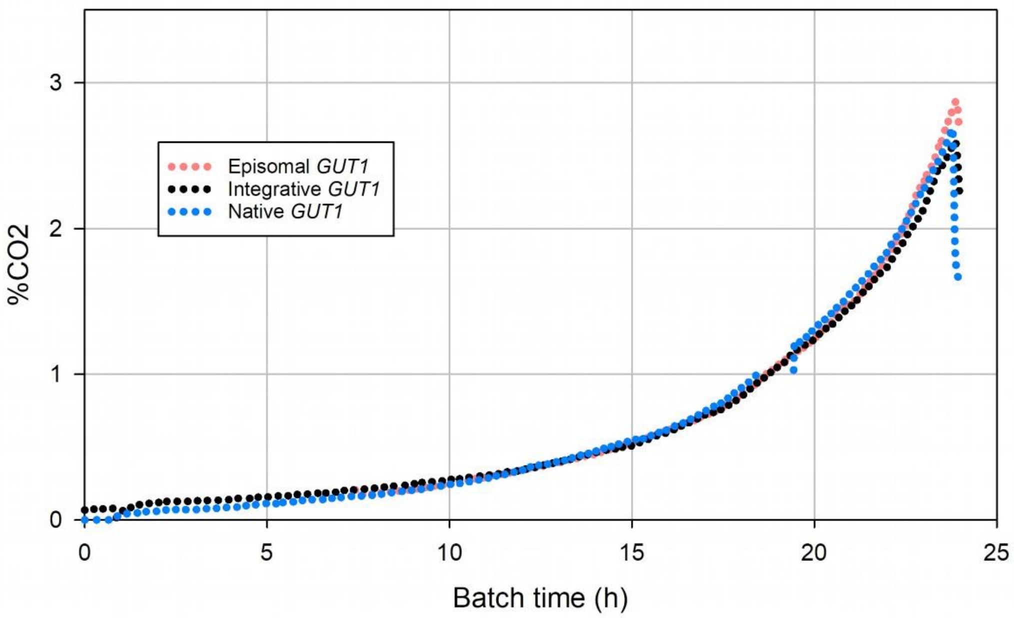

Figure S5: Maximum specific growth rates during the batch phase of a bioreactor cultivation based on off-gas analysis. Strains harbouring the native *GUT1* locus and episomal or integrative *GUT1* plasmids were analyzed.

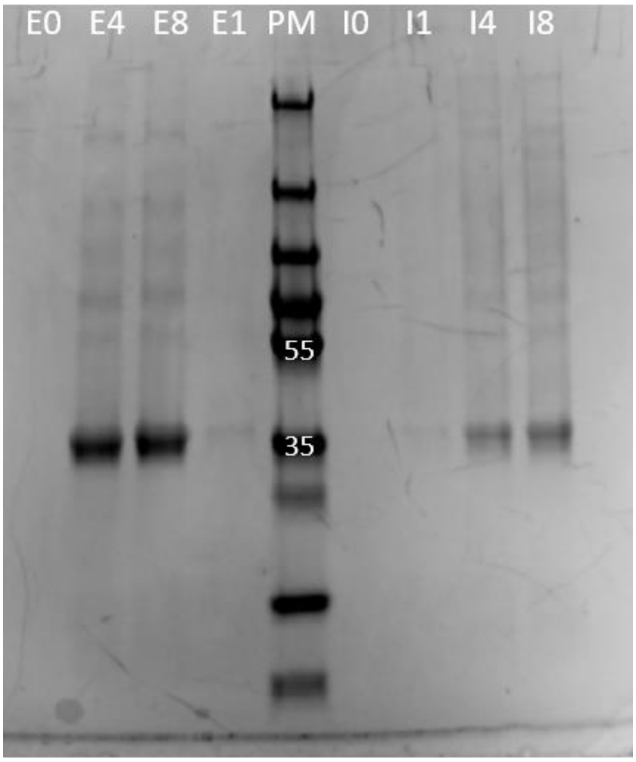

Figure S6. SDS page of bioreactor samples. 15 µl of bioreactor samples E0, E1, E4, E8, I0, I1, I4 and I8 were added to 5 µl of Laemmli Sample Buffer (Bio-Rad Laboratories, USA) and heated to 100 °C for 10 minutes. 10 µl of the sample preparations were loaded on a 5-15 % Mini-PROTEAN® TGX™ Precast Gel with 15 combs (Bio-Rad Laboratories, USA) together with the peqGOLD Protein Marker V (VWR International, USA) marked as PM in the image. The gel was stained with InstantBlue protein stain. The relevant 35 KDa and 55 KDa bands of the protein marker are indicated in the image to allow identification of the 33 KDa-sized *Ca*lB bands.

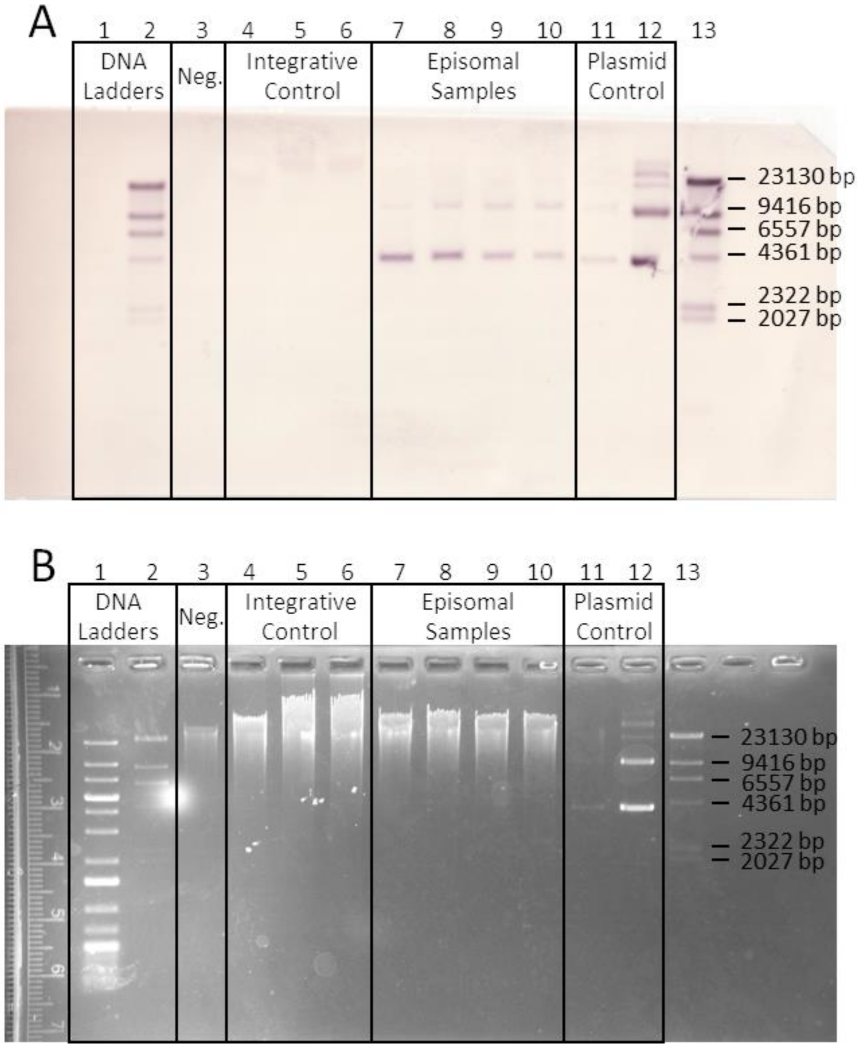

Figure S7. Southern Blot of *GUT*1-based ARS plasmid bioreactor cultivation. Genomic DNA was isolated from samples taken from the bioreactor cultivation of the episomal expression system as well as the integrative reference strain. Gel-electrophoresis (B), Southern blot and subsequent detection of the *Cal*B gene with a DIG-labeled probe (A) was performed. 1: Gene Ruler 1 kb plus not dig-labeled 0,5 µg; 2: DNA Molecular Weight Marker II, DIG-labeled (20 ng); 3: BSY11G1 empty strain; 4: BSY11G1 _FW001 A; 5: BSY11G1 _FW001 B; 6: BSY11G1 _FW001 C; 7: Bioreactor sample E0; 8: Bioreactor sample E1; 9: Bioreactor sample E4; 10: Bioreactor sample E8; 11: FW002 10 ng; 12: FW002 200 ng; 13: DNA Molecular Weight Marker II, DIG-labeled (20 ng).

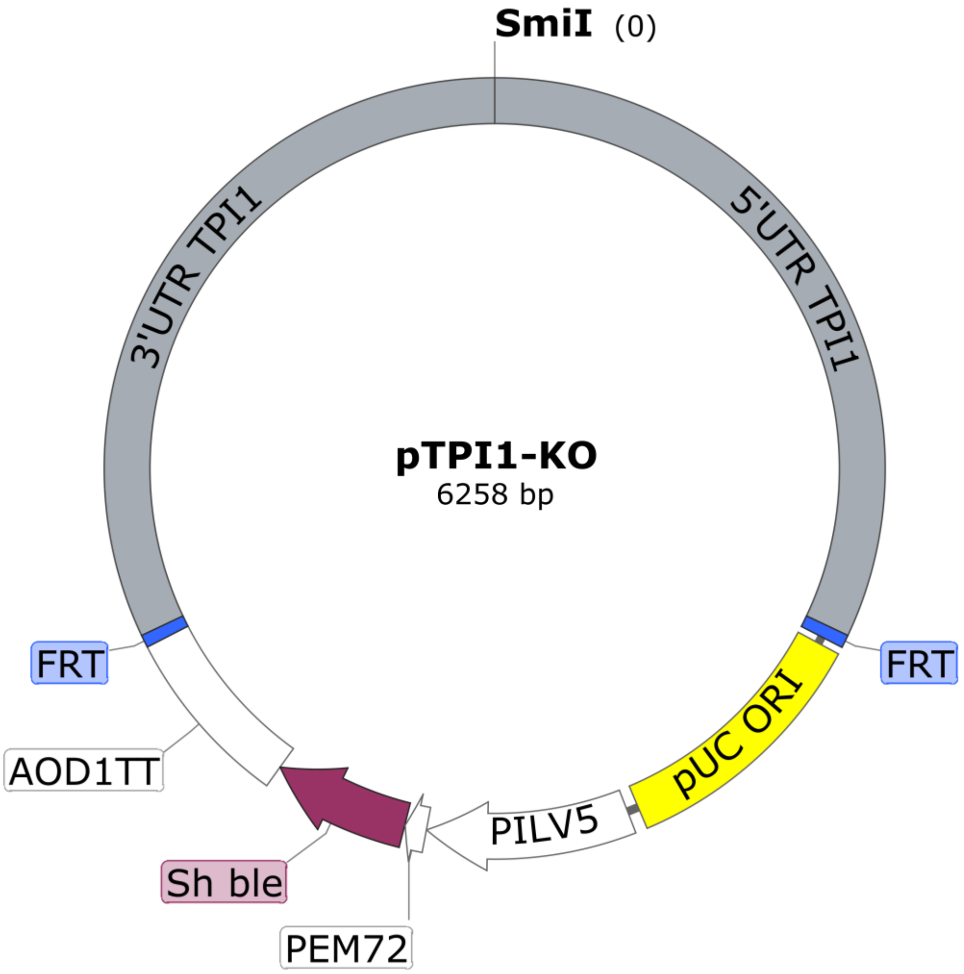

Figure S8. Vector map of the plasmid used for the deletion of *TPI1*. The shuttle vector shown in this figure harbors a Zeocin resistance cassette for both *E. coli* and *K. phaffii*, an origin of replication for *E. coli* and sequences homologous to the genomic sequences adjacent to the *TPI1* coding sequence in *K. phaffii*, in order to facilitate homologous recombination-based gene replacement and knock-out of the *TPI1* gene. The *Smi*I linearized plasmid was used to transform *K. phaffii* according to Lin-Cereghino *et al.* (2005). The transformation was plated on agar plates containing 2 % (w/v) peptone, 1 % (w/v) yeast extract, 0.1 % (w/v) glucose, 2 % (v/v) ethanol, 1.5 (w/v) % agar and 100 mg/L Zeocin and was incubated at 28 °C for 7-10 days.

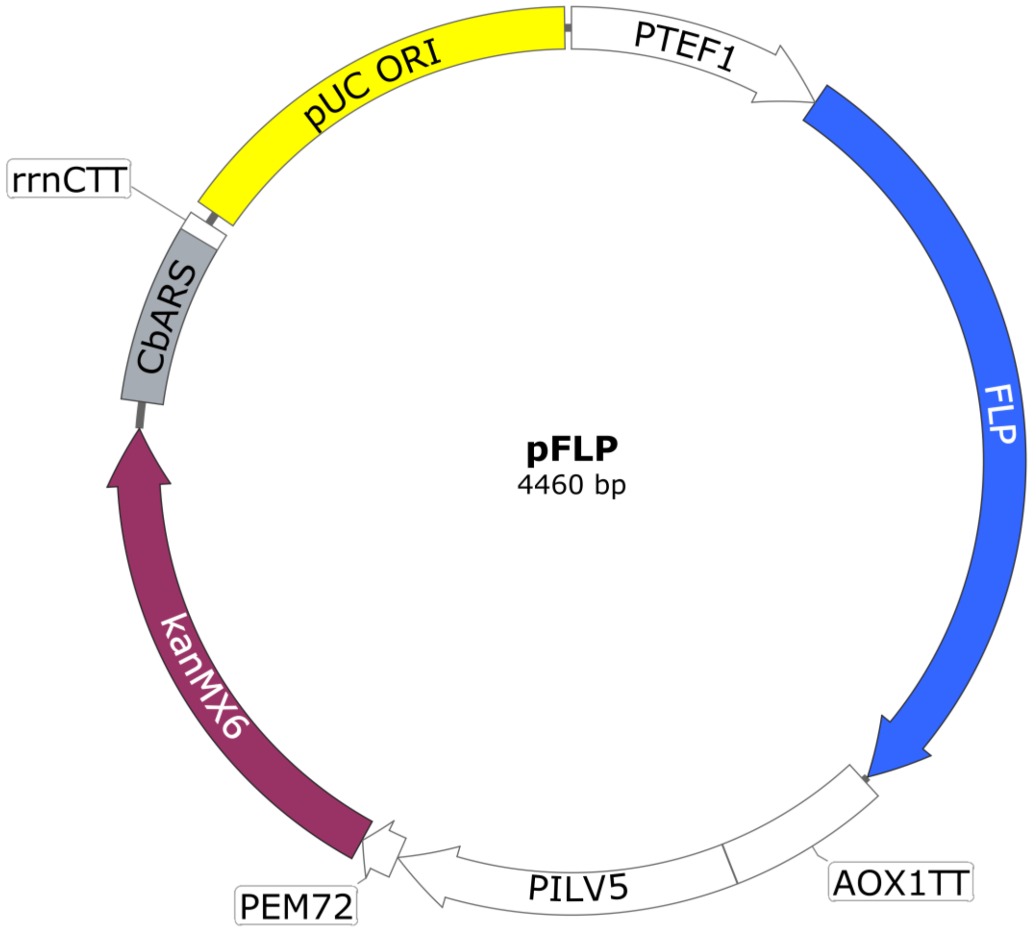

Figure S9. Vector map of the plasmid used for marker recycling in the *TPI1* knock-out strain. The shuttle vector shown in this figure harbors a Kanamycin/Geneticin resistance cassette, an origin of replication and the *Cb*ARS sequence for the replication in both *E. coli* and *K. Phaffii*, respectively. Additionally, a flippase expression cassette with the constitutive *TEF1* promoter is included, too. This plasmid was used to transform *TPI1* knock-out strains in order to recycle the Zeocin resistance marker with the Flp/FRT system.

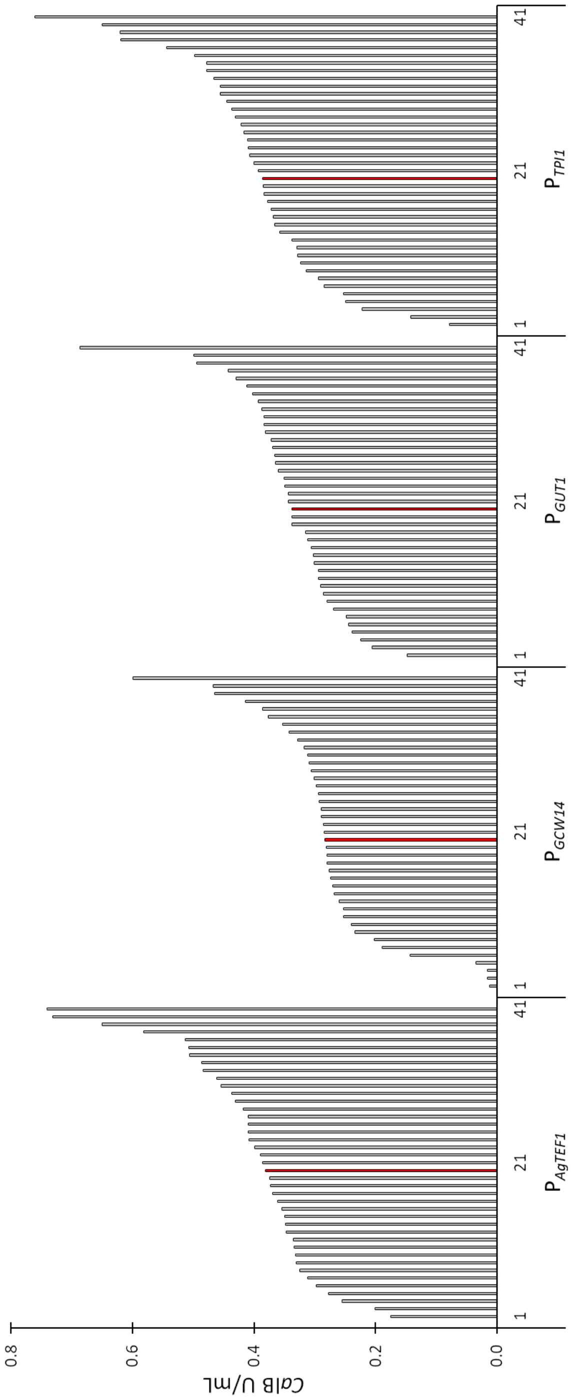

Figure S10. Median Screening of integrative *GUT1* plasmid transformants. *K. phaffii* BSY11G1 was transformed with *Smi*I linearized plasmids 5, 7, 9, 11 and 13 according to Lin-Cereghino *et al.* (2005). 41 transformants of each transformation (no transformants obtained for plasmid 11) were cultivated in 96 deep-well plates and the *Cal*B activity was determined. The median clone of each plasmid was used as reference strain for the respective episomal plasmid in following experiments.

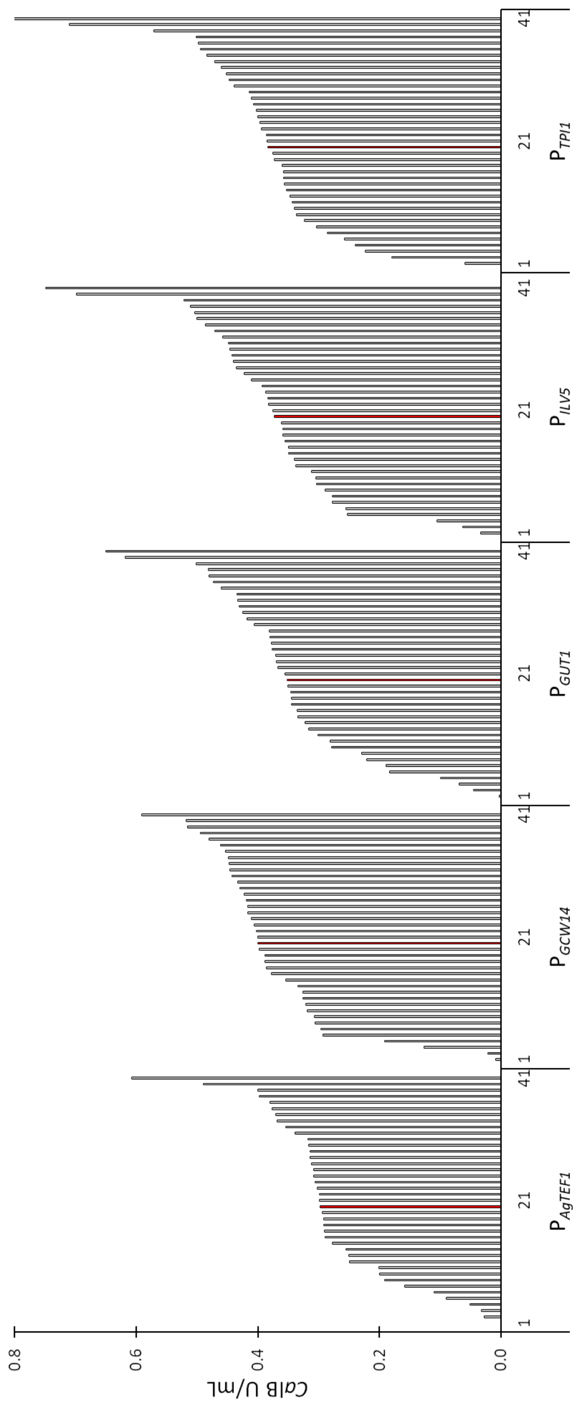

Figure S11. Median Screening of integrative *TPI1* plasmid transformants. *K. phaffii* BSYBG11 Δ*tpi1* was transformed with *Smi*I linearized plasmids 15, 17, 19, 21 and 23 according to Lin-Cereghino *et al.* (2005). 41 transformants of each transformation were cultivated in 96 deep-well plates and the *Cal*B activity was determined. The median clone of each plasmid was used as reference strain for the respective episomal plasmid in following experiments.
