# Supplementary material for: Scalable protein production by Komagataella phaffii enabled by ARS plasmids and carbon source-based selection
Source: Microb Cell Fact. 2024 Apr 20;23:116. doi: 10.1186/s12934-024-02368-3 (PMC11031860; doi:10.1186/s12934-024-02368-3)

Qualimap Analysis Results

BAM QC analysis

Generated by Qualimap v.2.2.1

2024/03/10 14:35:31

1. Input data & parameters

1.1. QualiMap command line

qualimap bamqc -bam /workdir/FW001Median/FW001Median.dups_rem.bam -c -nw 400 -hm 3 -sd

1.2. Alignment

Command line:	bwa mem -t 16 -a -M -R @RG\tID:pichia\tSM:FW001Median\tPL:ILLUMINA /workdir/new_reference/CBS7435_Schaffer_plasmids.fasta /workdir/trimmed/FW001Median_1_trimmed.fastq /workdir/trimmed/FW001Median_2_trimmed.fastq
Draw chromosome limits:	yes
Analyze overlapping paired-end reads:	no
Program:	bwa (0.7.17-r1188)
Analysis date:	Sun Mar 10 14:35:31 MSK 2024
Size of a homopolymer:	3
Skip duplicate alignments:	yes (only flagged)
Number of windows:	400
BAM file:	/workdir/FW001Median/FW001Median.dups_rem.bam

2. Summary

2.1. Globals

Reference size	9,447,793
Number of reads	21,497,431
Mapped reads	21,472,968 / 99.89%
Unmapped reads	24,463 / 0.11%
Mapped paired reads	21,472,968 / 99.89%
Mapped reads, first in pair	10,745,414 / 49.98%
Mapped reads, second in pair	10,727,554 / 49.9%
Mapped reads, both in pair	21,446,800 / 99.76%
Mapped reads, singletons	26,168 / 0.12%
Read min/max/mean length	0 / 151 / 147.89
Duplicated reads (flagged)	3,399,018 / 15.81%
Clipped reads	164,848 / 0.77%
Duplicated reads skipped:	3,399,018 / 15.81%

2.2. ACGT Content

Number/percentage of A's	827,528,725 / 30.94%
Number/percentage of C's	509,295,283 / 19.04%
Number/percentage of T's	813,893,674 / 30.43%
Number/percentage of G's	523,682,512 / 19.58%
Number/percentage of N's	53,303 / 0%
GC Percentage	38.62%

2.3. Coverage

Mean	283.087
Standard Deviation	628.4238

2.4. Mapping Quality

Mean Mapping Quality	58.76

2.5. Insert size

Mean	1,755.99
Standard Deviation	45,151.3
P25/Median/P75	519 / 608 / 710

2.6. Mismatches and indels

General error rate	0.29%
Mismatches	7,601,730
Insertions	74,860
Mapped reads with at least one insertion	0.31%
Deletions	25,824
Mapped reads with at least one deletion	0.1%
Homopolymer indels	65.08%

2.7. Chromosome stats

**Name**	**Length**	**Mapped bases**	**Mean coverage**	**Standard deviation**

LT962476.2	2895357	720531364	248.8575	146.7532
LT962477.2	2396459	575297505	240.0615	26.6968
LT962478.2	2263464	556576362	245.8958	99.8206
LT962479.2	1827946	452192496	247.3774	105.7108
FR839632.1	35683	368424008	10,324.9169	552.5339
MG491503.1	13092	0	0	0
MG491504.1	9448	151	0.016	0.1254
Linear	6344	1525323	240.4355	32.7903

3. Results : Coverage across reference

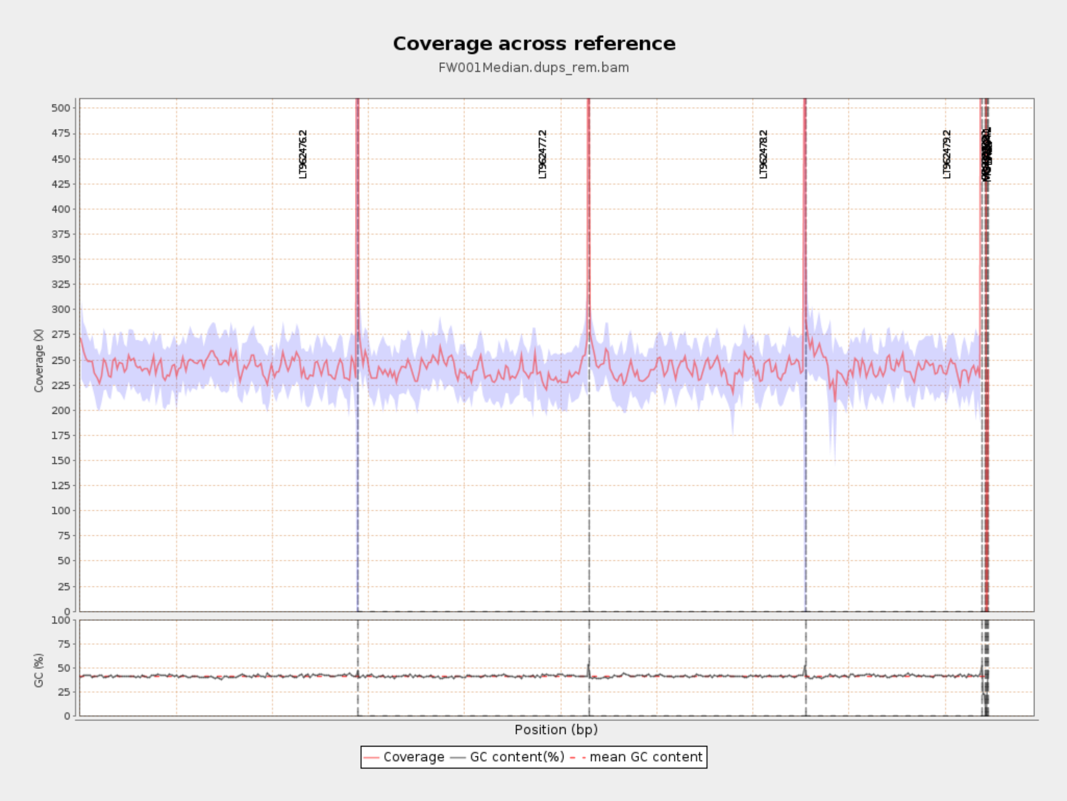

4. Results : Coverage Histogram

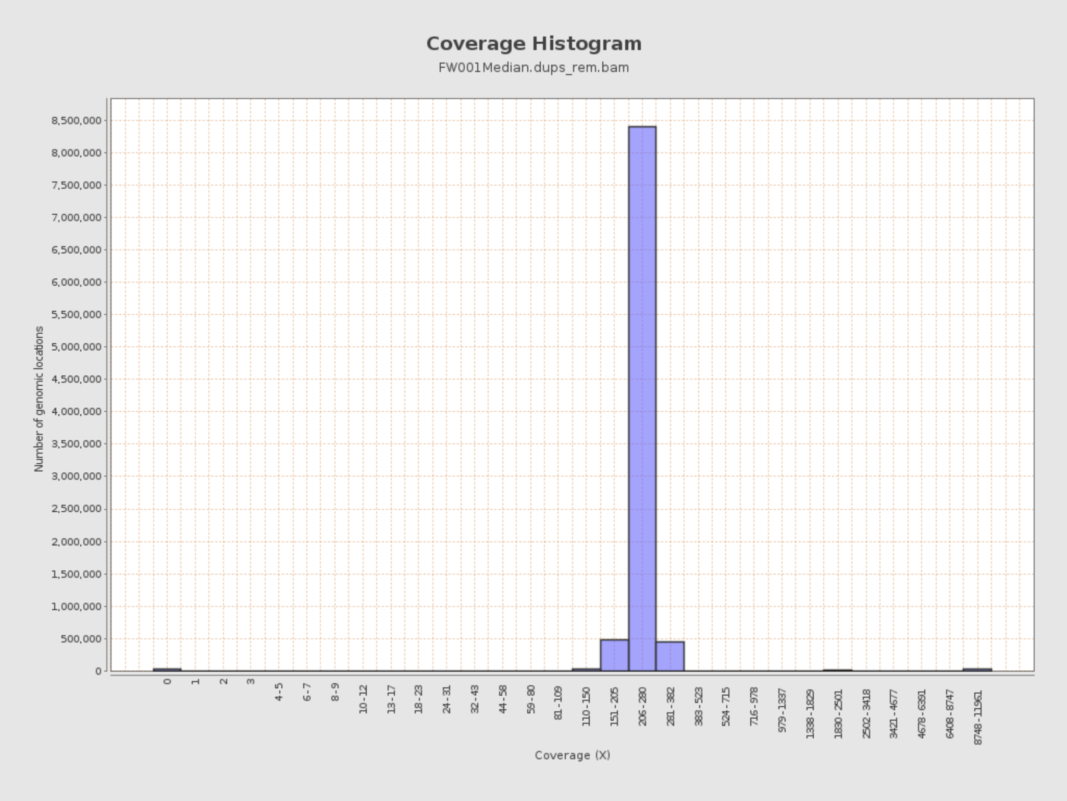

5. Results : Coverage Histogram (0-50X)

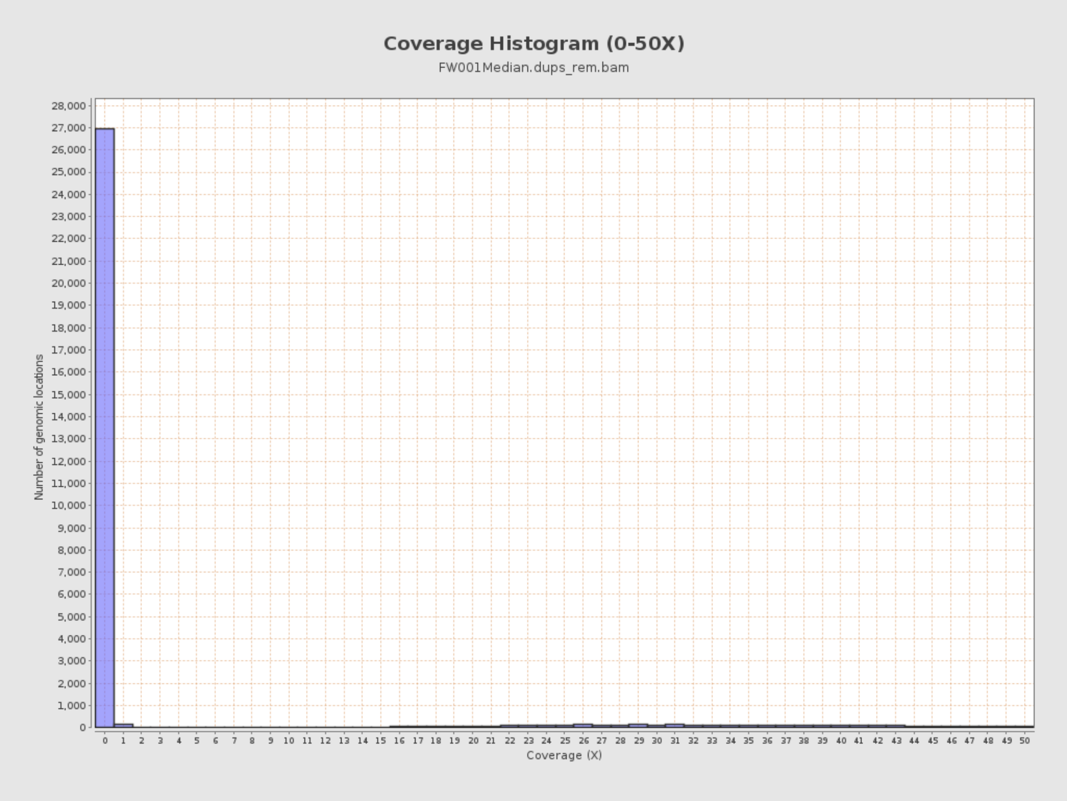

6. Results : Genome Fraction Coverage

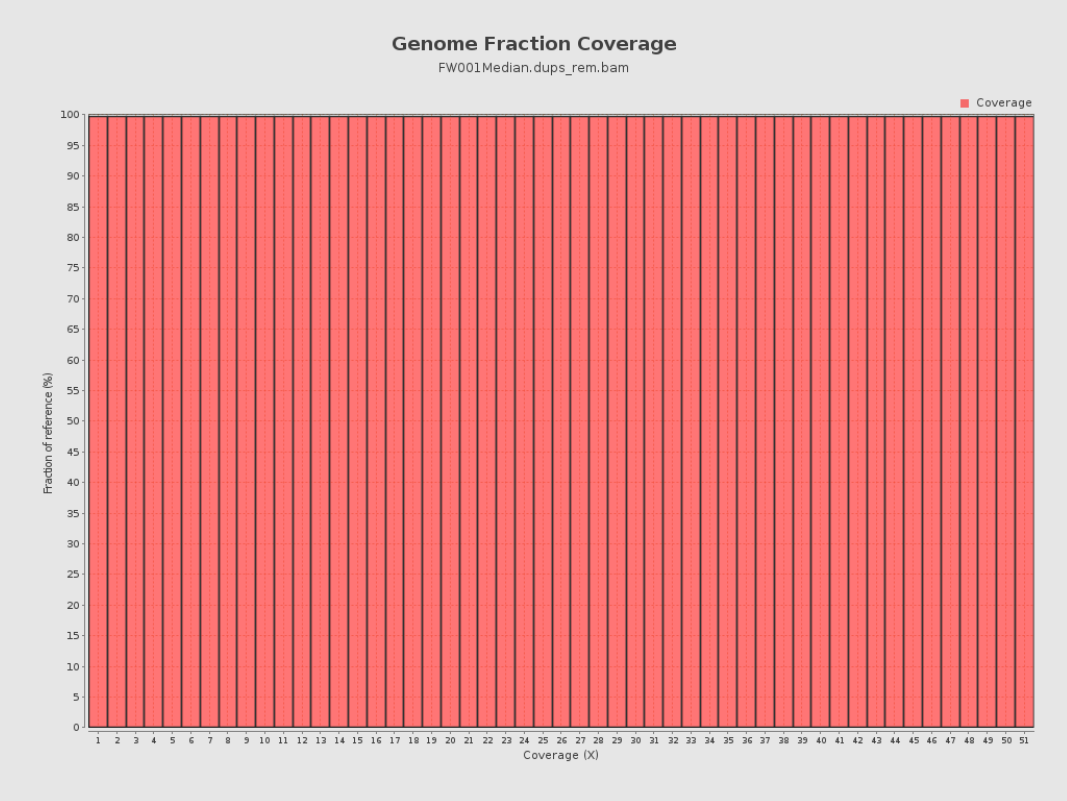

7. Results : Duplication Rate Histogram

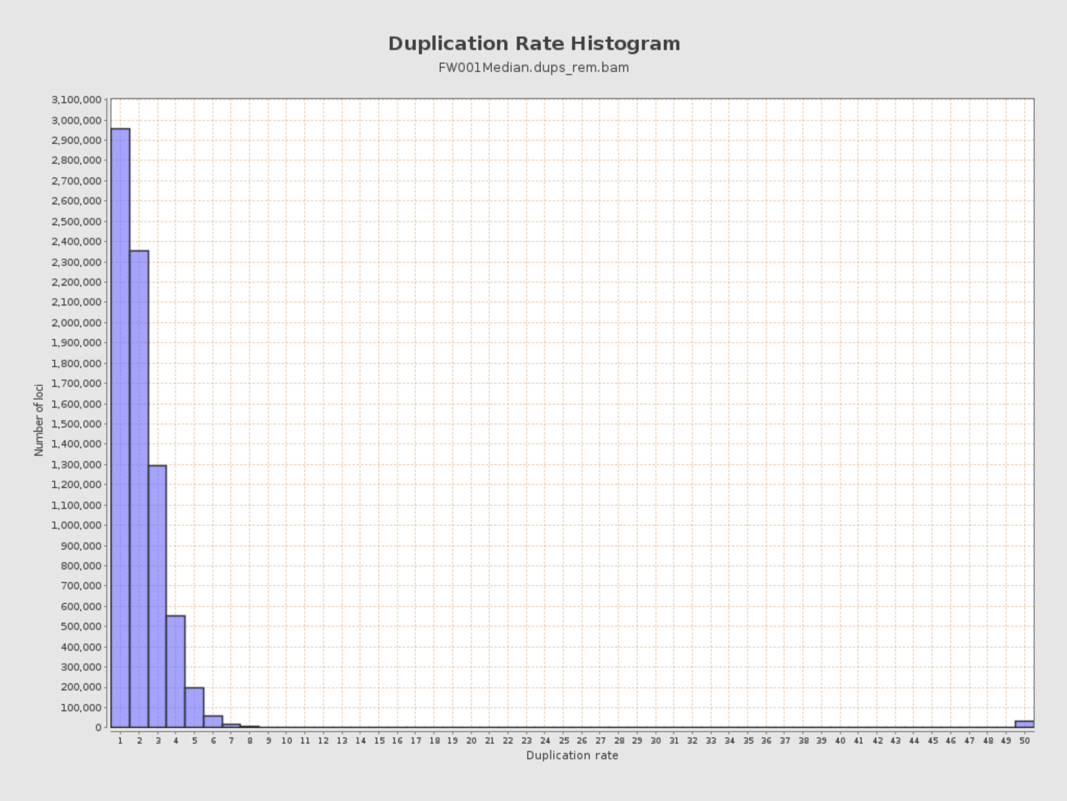

8. Results : Mapped Reads Nucleotide Content

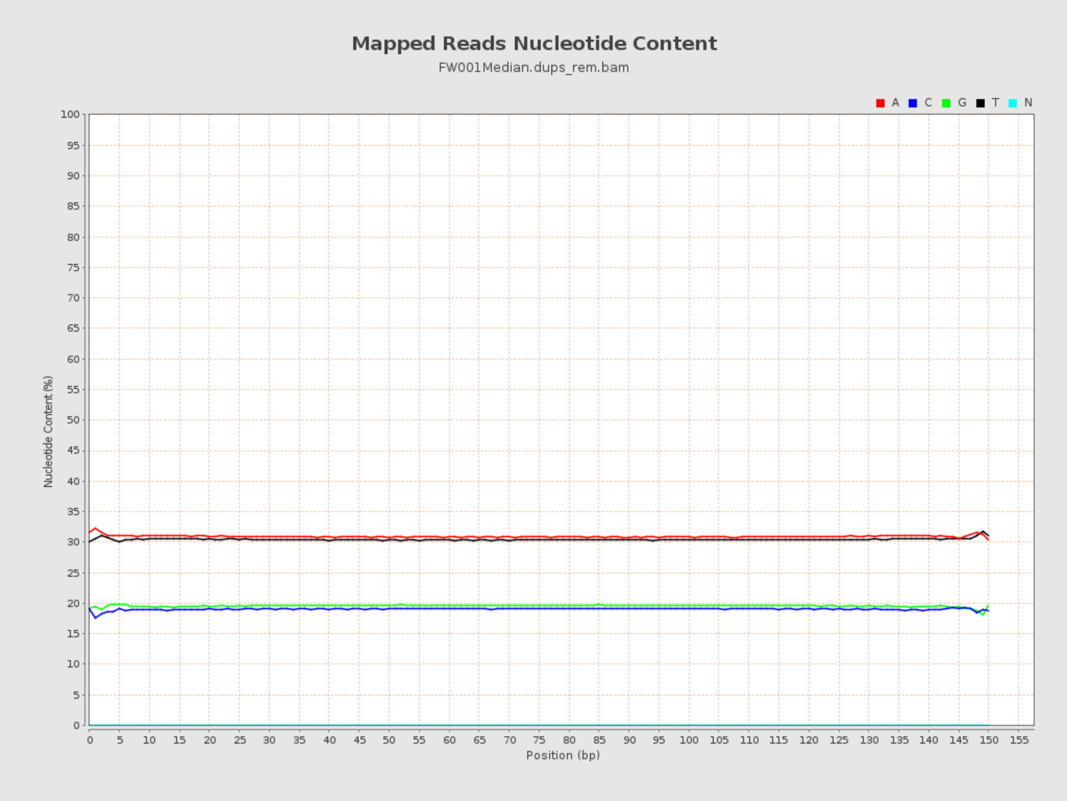

9. Results : Mapped Reads GC-content Distribution

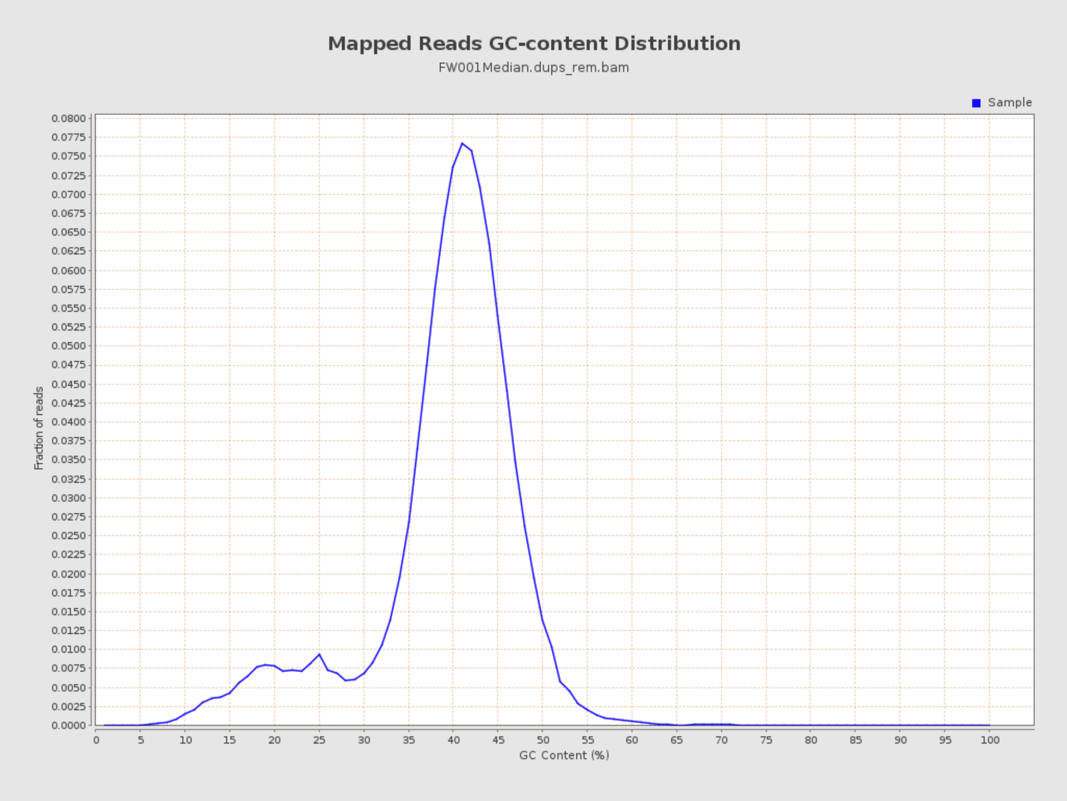

10. Results : Mapped Reads Clipping Profile

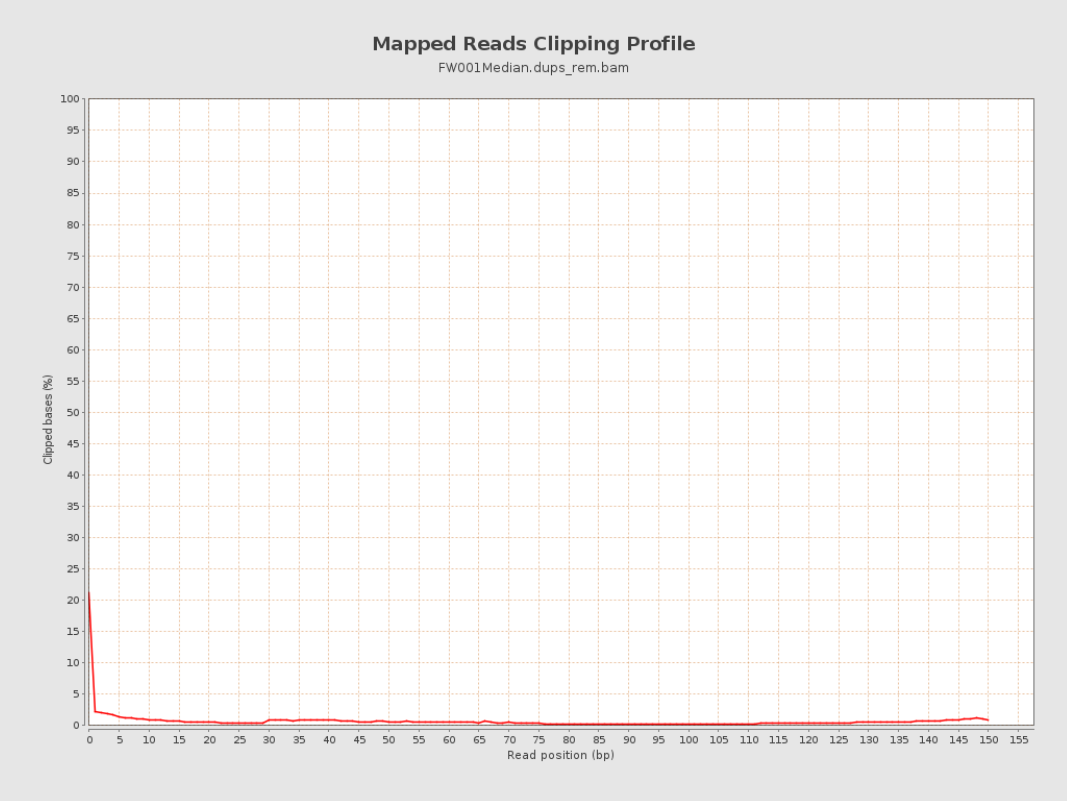

11. Results : Homopolymer Indels

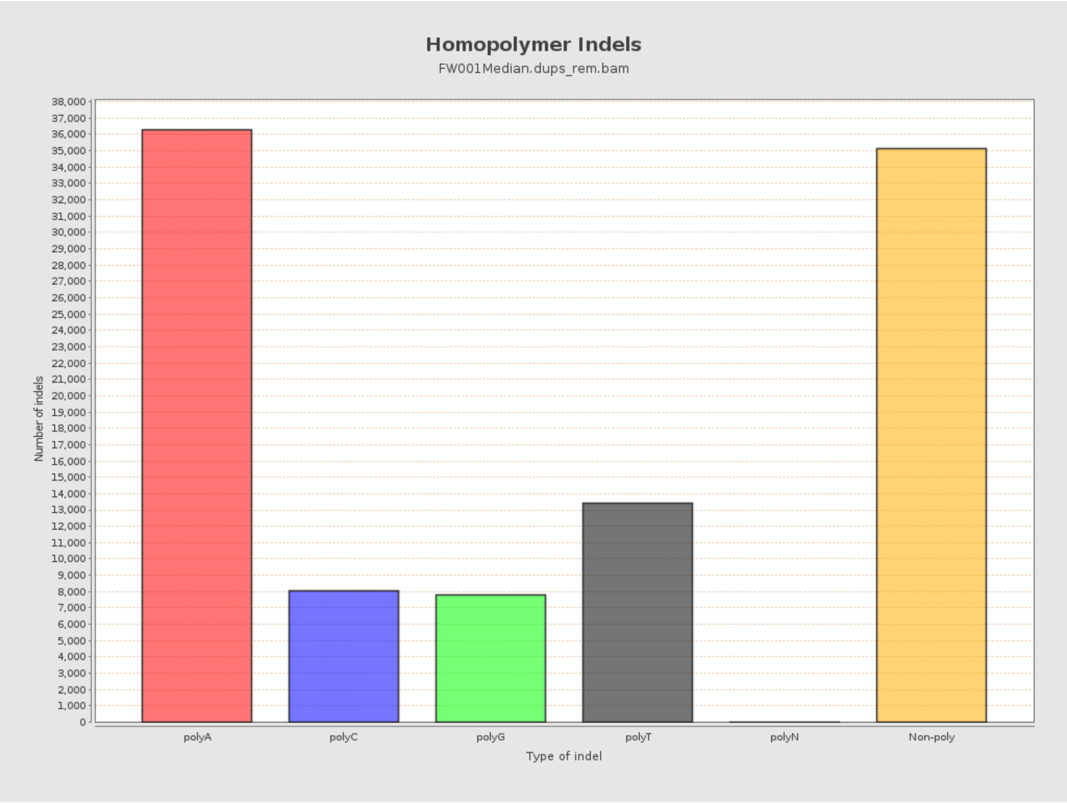

12. Results : Mapping Quality Across Reference

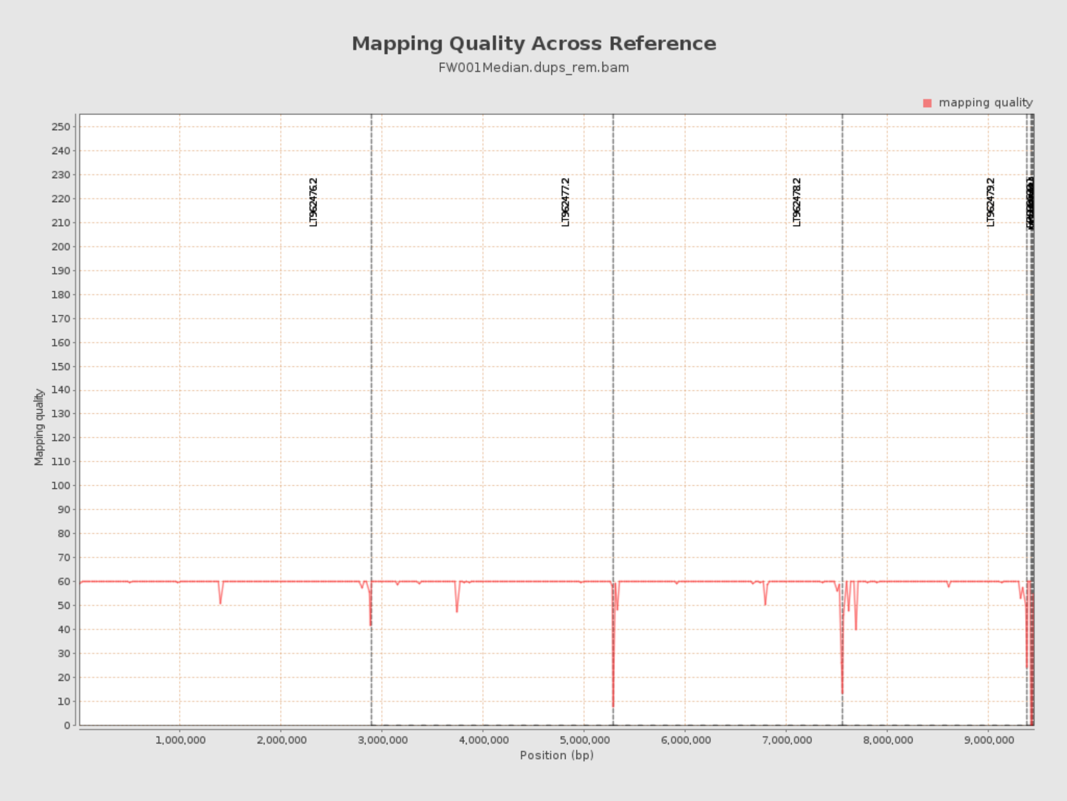

13. Results : Mapping Quality Histogram

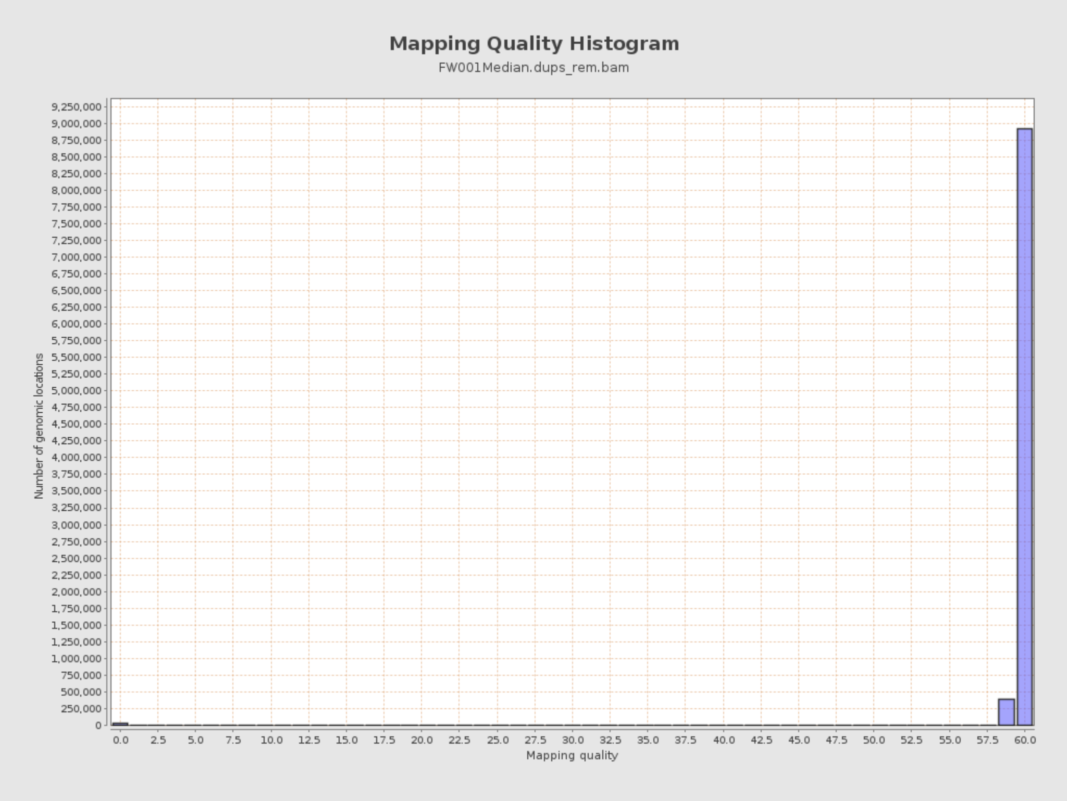

14. Results : Insert Size Across Reference

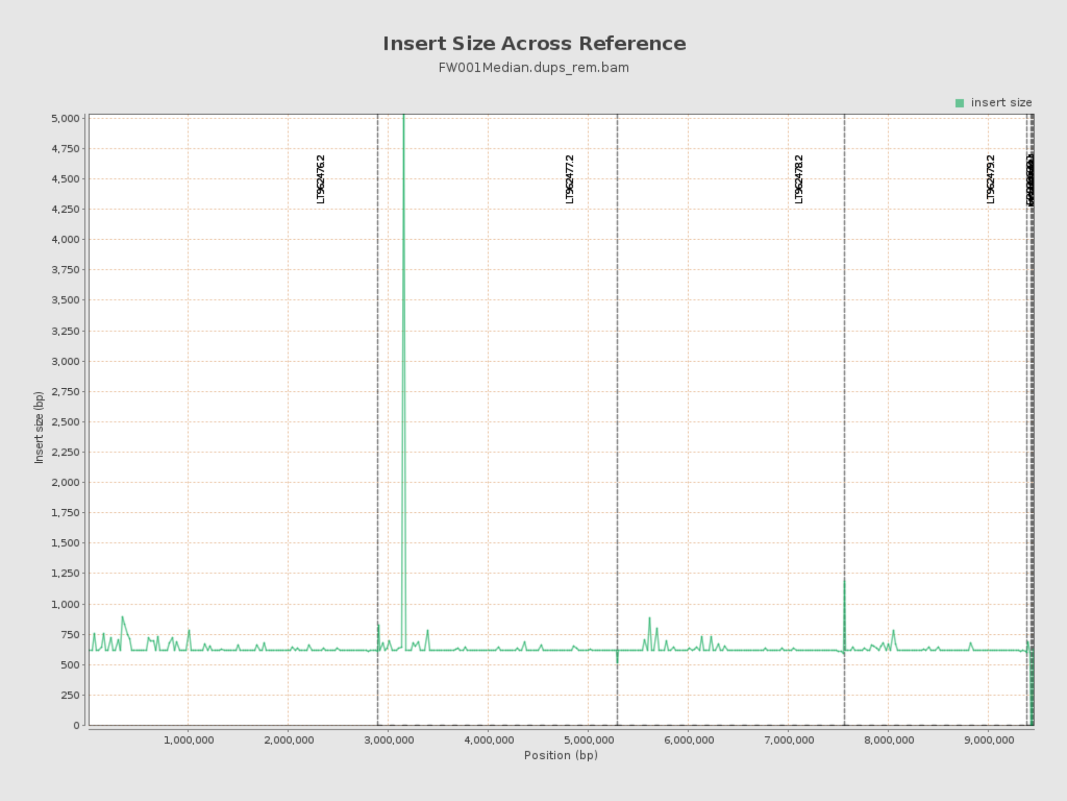

15. Results : Insert Size Histogram

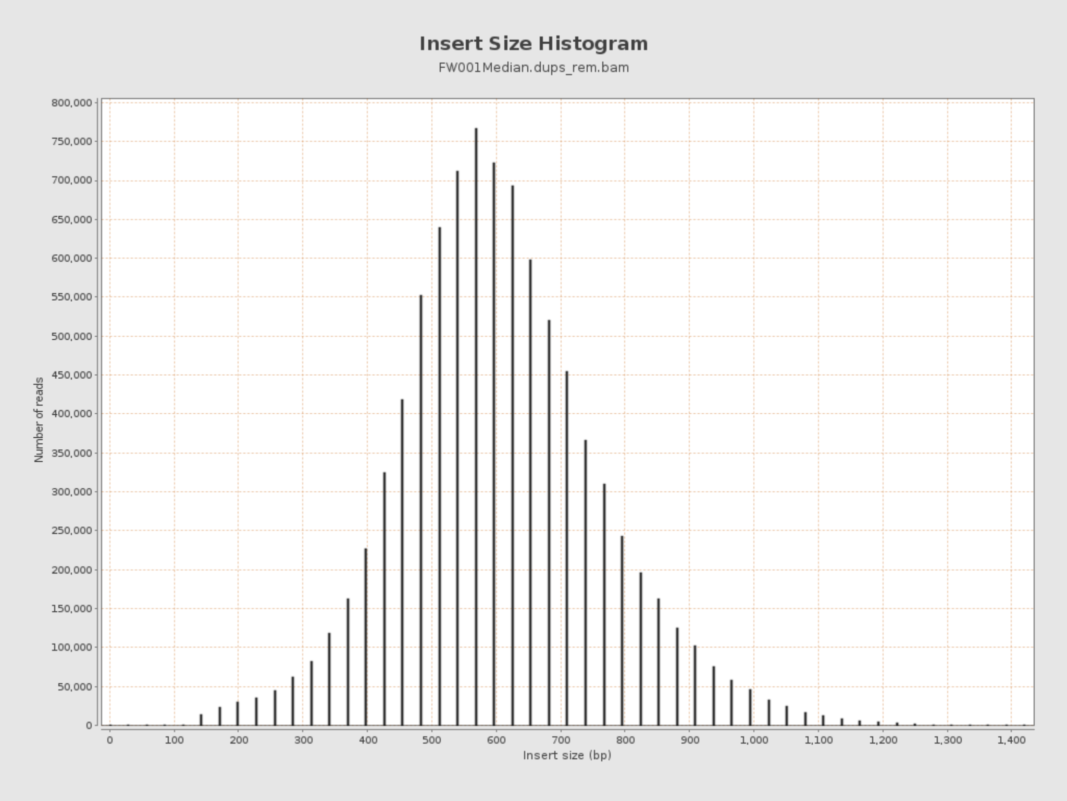

Supplement: Supplementary file 2 — Supplementary Material 2 [file 12934_2024_2368_MOESM2_ESM.zip › Supplementary_File_2/BSYBG11gut1_integrative_reference/FW001Median_qualimap.pdf]
