# Supplementary material for: Scalable protein production by Komagataella phaffii enabled by ARS plasmids and carbon source-based selection
Source: Microb Cell Fact. 2024 Apr 20;23:116. doi: 10.1186/s12934-024-02368-3 (PMC11031860; doi:10.1186/s12934-024-02368-3)

Qualimap Analysis Results

BAM QC analysis

Generated by Qualimap v.2.2.1

2023/04/27 14:45:21

1. Input data & parameters

1.1. QualiMap command line

qualimap bamqc -bam /workdir/BG11tpi-FRT1_1/BG11tpi-FRT1_1.dups_rem.bam -c -nw 400 -hm 3 -sd

1.2. Alignment

Command line:	bwa mem -t 16 -a -M -R @RG\tID:pichia\tSM:BG11tpi-FRT1_1\tPL:ILLUMINA /workdir/new_reference/CBS7435_Schaffer_plasmids.fasta /workdir/trimmed/BG11tpi-FRT1_1_1_trimmed.fastq /workdir/trimmed/BG11tpi-FRT1_1_2_trimmed.fastq
Draw chromosome limits:	yes
Analyze overlapping paired-end reads:	no
Program:	bwa (0.7.17-r1188)
Analysis date:	Thu Apr 27 14:45:21 MSK 2023
Size of a homopolymer:	3
Skip duplicate alignments:	yes (only flagged)
Number of windows:	400
BAM file:	/workdir/BG11tpi-FRT1_1/BG11tpi-FRT1_1.dups_rem.bam

2. Summary

2.1. Globals

Reference size	9,448,159
Number of reads	10,271,787
Mapped reads	10,239,112 / 99.68%
Unmapped reads	32,675 / 0.32%
Mapped paired reads	10,239,112 / 99.68%
Mapped reads, first in pair	5,135,664 / 50%
Mapped reads, second in pair	5,103,448 / 49.68%
Mapped reads, both in pair	10,204,447 / 99.34%
Mapped reads, singletons	34,665 / 0.34%
Read min/max/mean length	0 / 151 / 136.77
Duplicated reads (flagged)	844,481 / 8.22%
Clipped reads	306,231 / 2.98%
Duplicated reads skipped:	844,481 / 8.22%

2.2. ACGT Content

Number/percentage of A's	389,984,287 / 30.3%
Number/percentage of C's	253,605,259 / 19.71%
Number/percentage of T's	385,720,424 / 29.97%
Number/percentage of G's	257,600,649 / 20.02%
Number/percentage of N's	9,162 / 0%
GC Percentage	39.72%

2.3. Coverage

Mean	136.2126
Standard Deviation	181.3285

2.4. Mapping Quality

Mean Mapping Quality	58.45

2.5. Insert size

Mean	1,018.08
Standard Deviation	27,881.98
P25/Median/P75	515 / 596 / 687

2.6. Mismatches and indels

General error rate	1.01%
Mismatches	12,870,164
Insertions	28,091
Mapped reads with at least one insertion	0.24%
Deletions	10,800
Mapped reads with at least one deletion	0.1%
Homopolymer indels	56.56%

2.7. Chromosome stats

Name	Length	Mapped bases	Mean coverage	Standard deviation

LT962476.2	2895357	368267957	127.1926	71.328
LT962477.2	2396459	297825292	124.2772	17.5942
LT962478.2	2263464	285048324	125.9346	40.5078
LT962479.2	1827946	230283634	125.9795	44.2801
FR839632.1	35683	105518486	2,957.108	259.4235
MG491503.1	13092	0	0	0
MG491504.1	9448	0	0	0
FW049_pTPI-FRT-K	2250	6060	2.6933	11.8627
FW050_pBSYA2K_FL	4460	8291	1.859	8.7028

3. Results : Coverage across reference

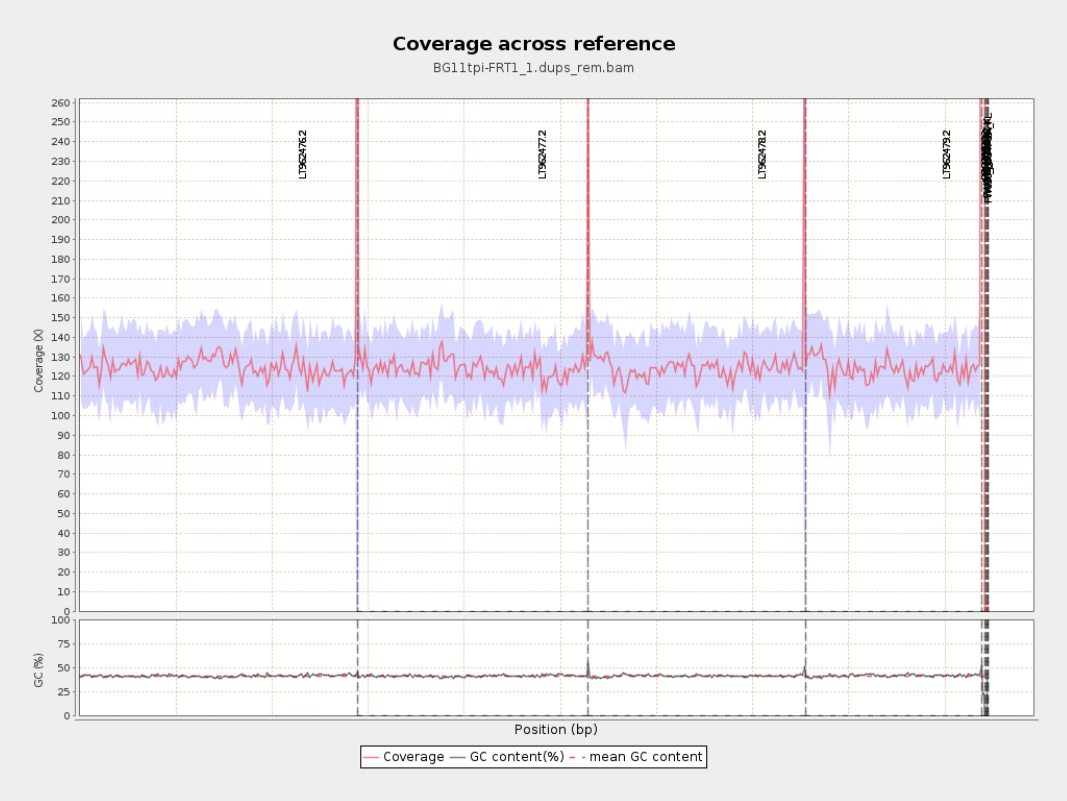

4. Results : Coverage Histogram

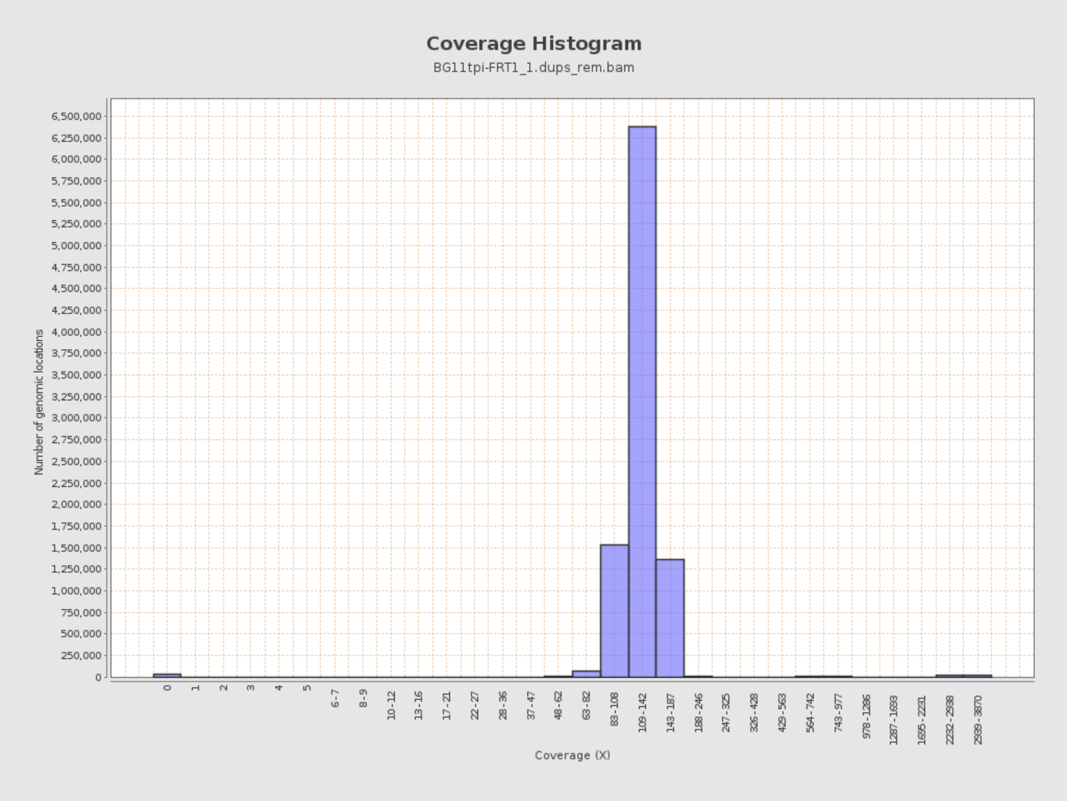

5. Results : Coverage Histogram (0-50X)

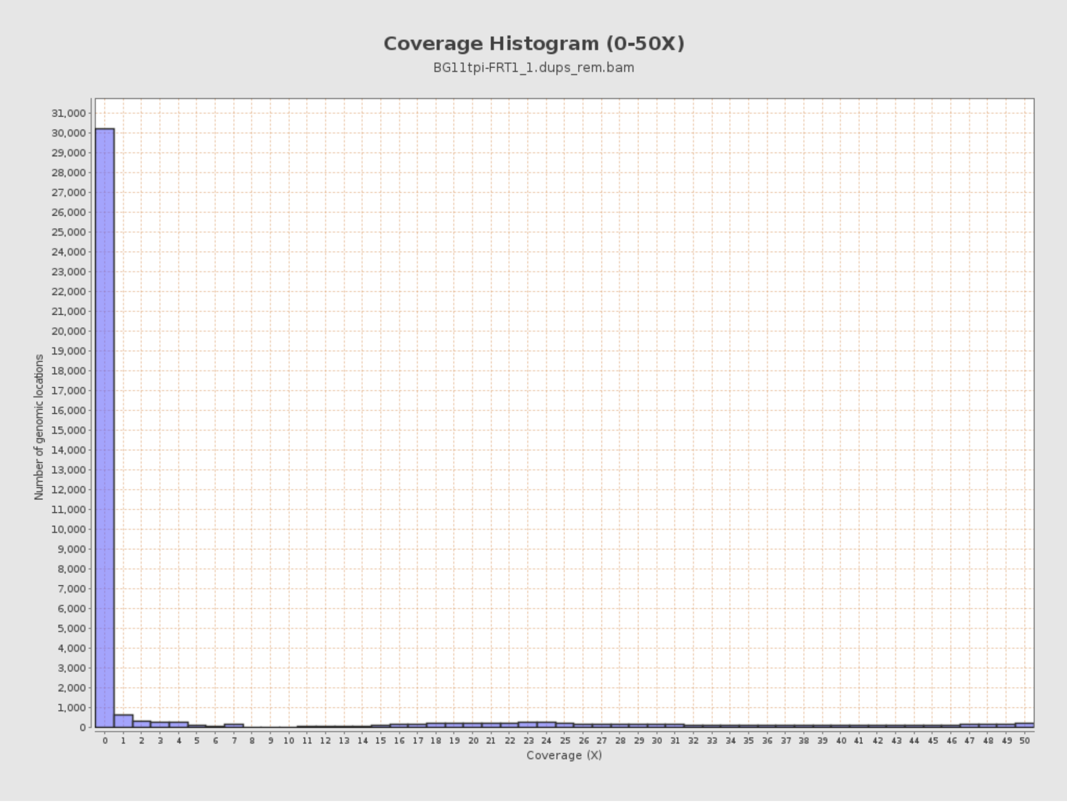

6. Results : Genome Fraction Coverage

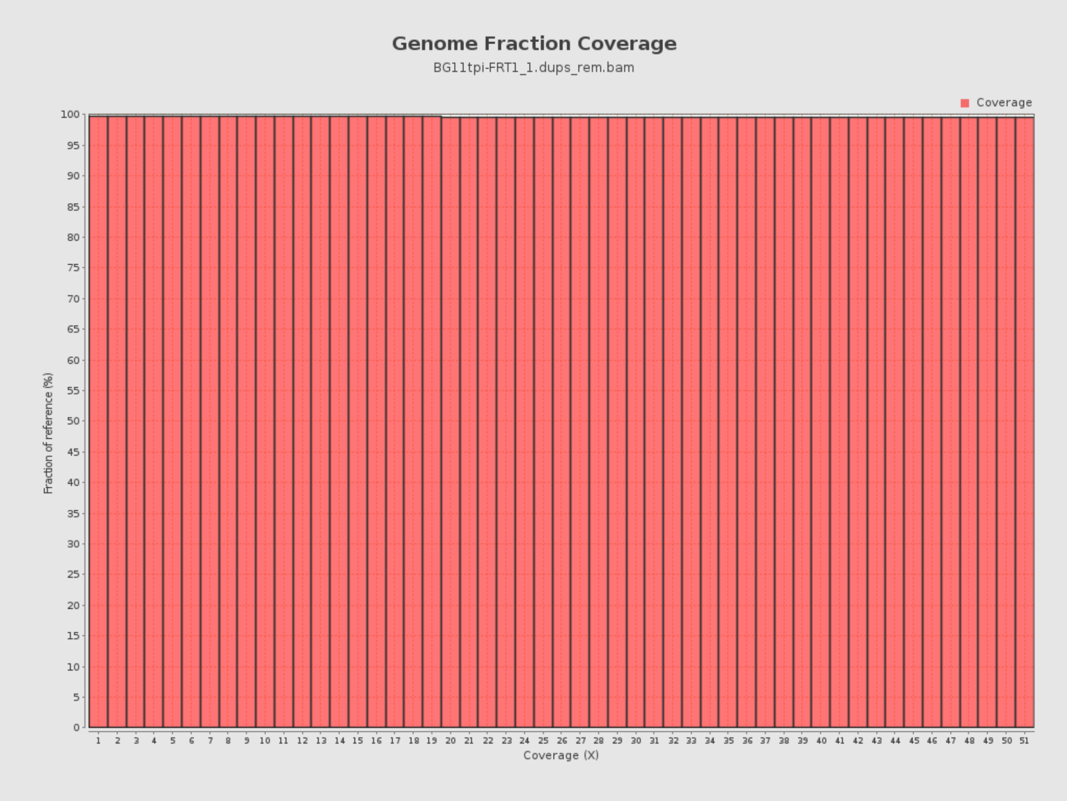

7. Results : Duplication Rate Histogram

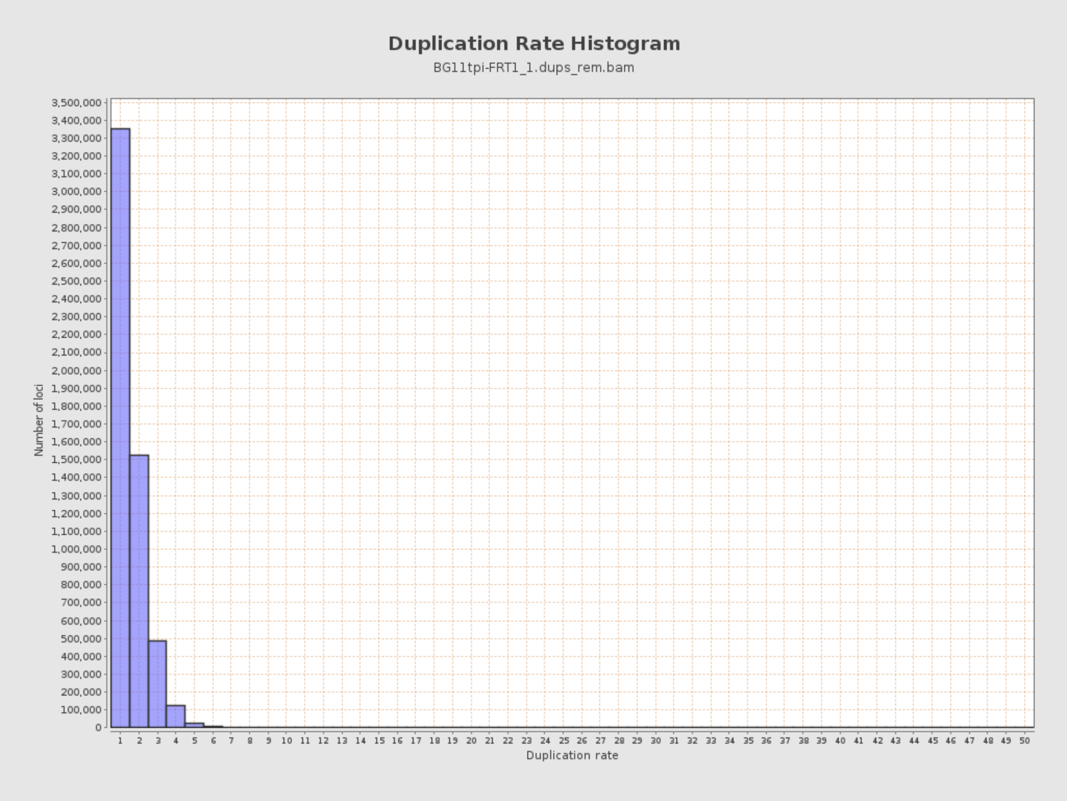

8. Results : Mapped Reads Nucleotide Content

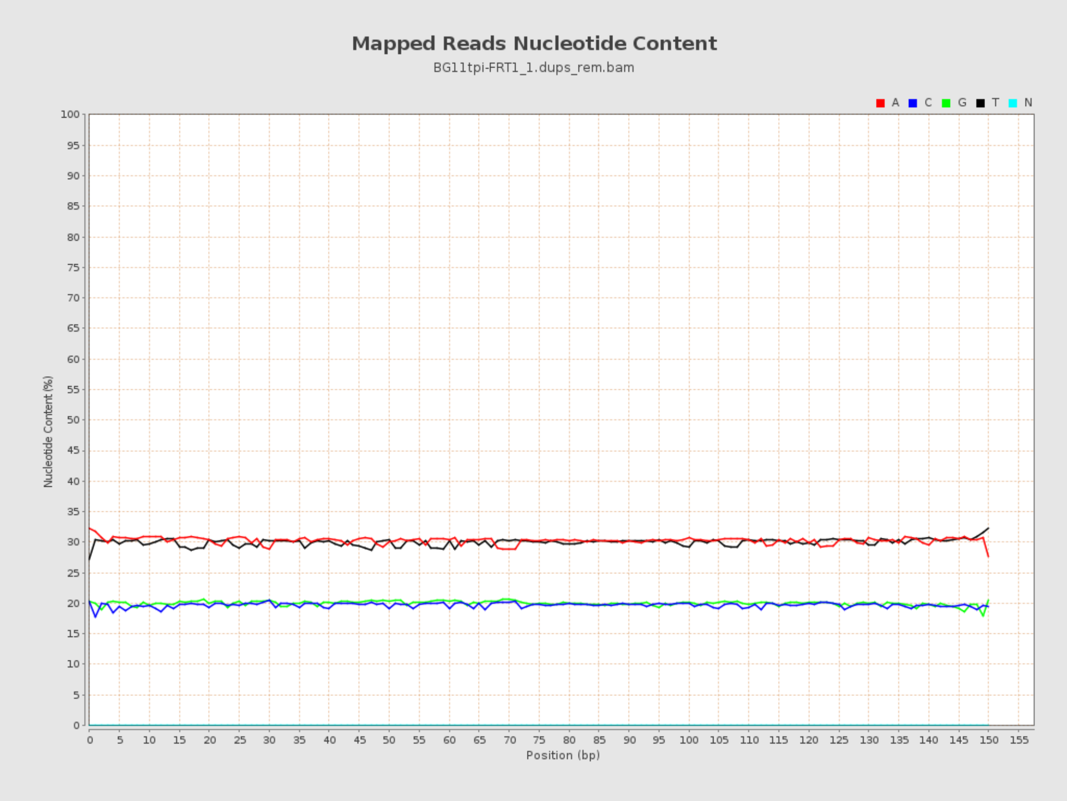

9. Results : Mapped Reads GC-content Distribution

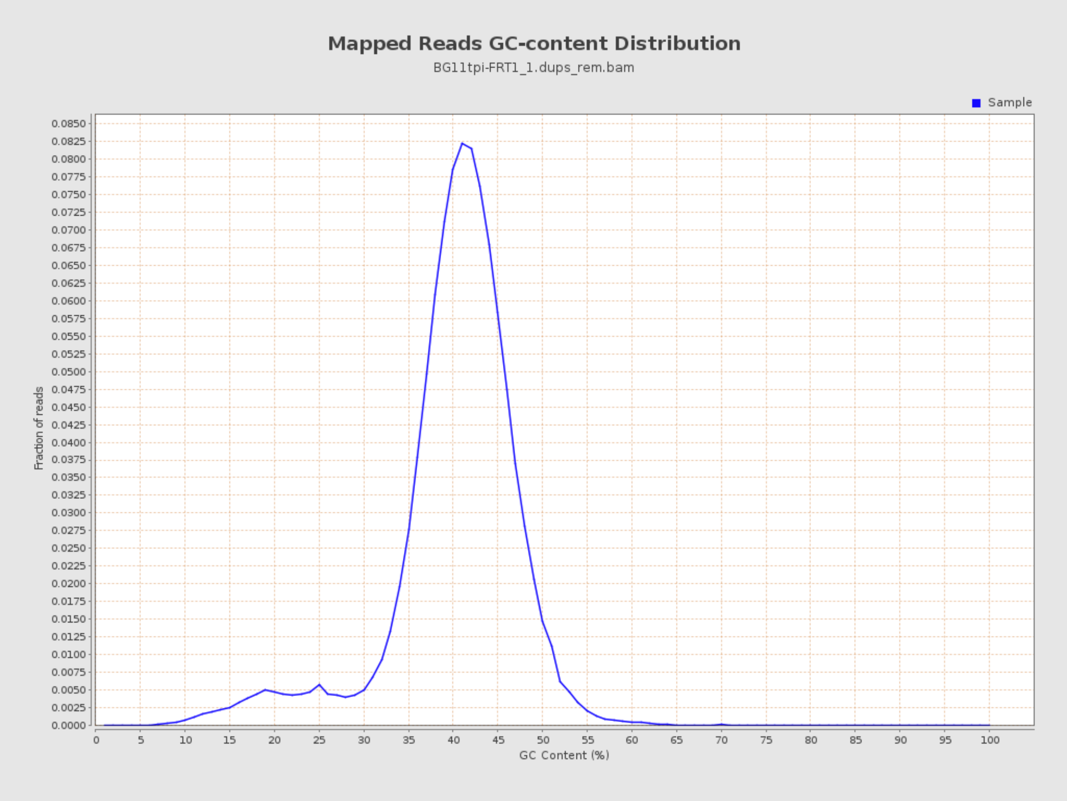

10. Results : Mapped Reads Clipping Profile

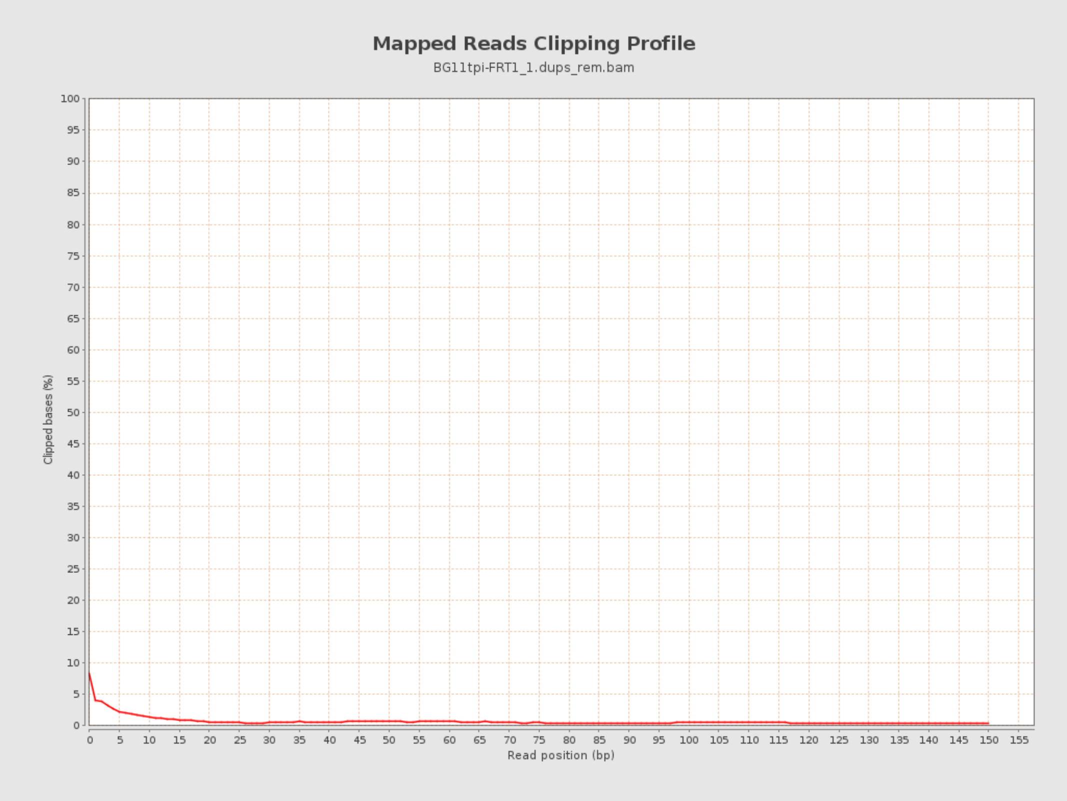

11. Results : Homopolymer Indels

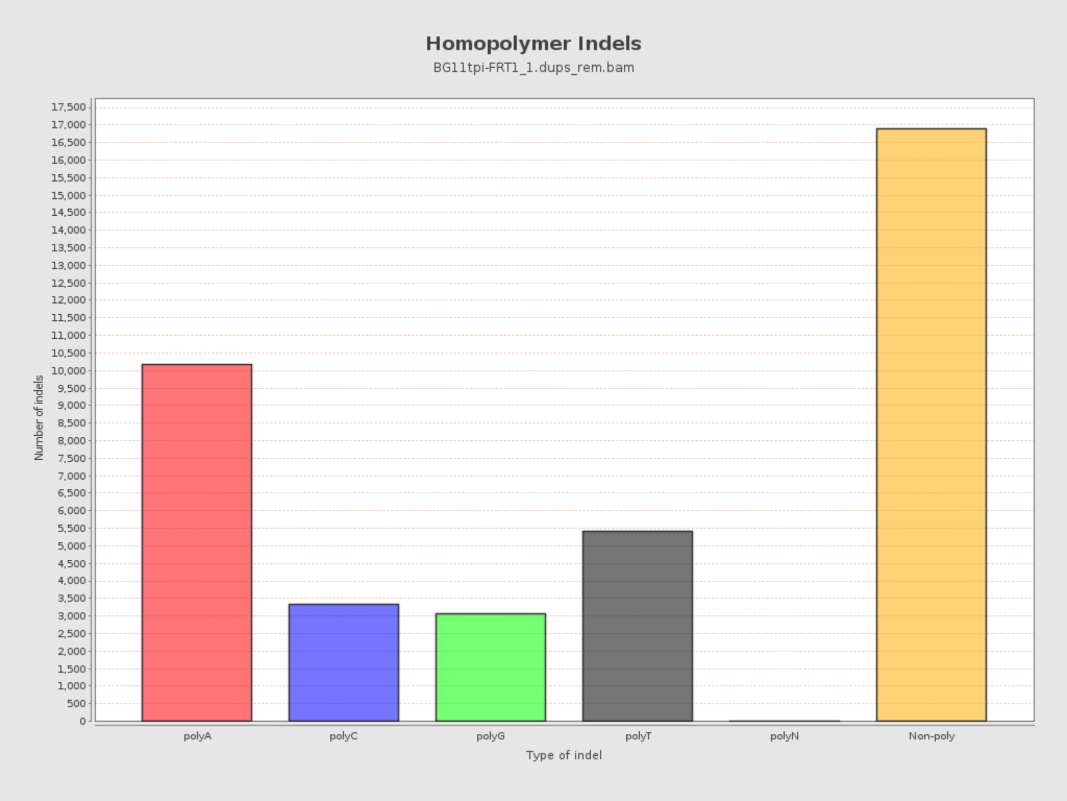

12. Results : Mapping Quality Across Reference

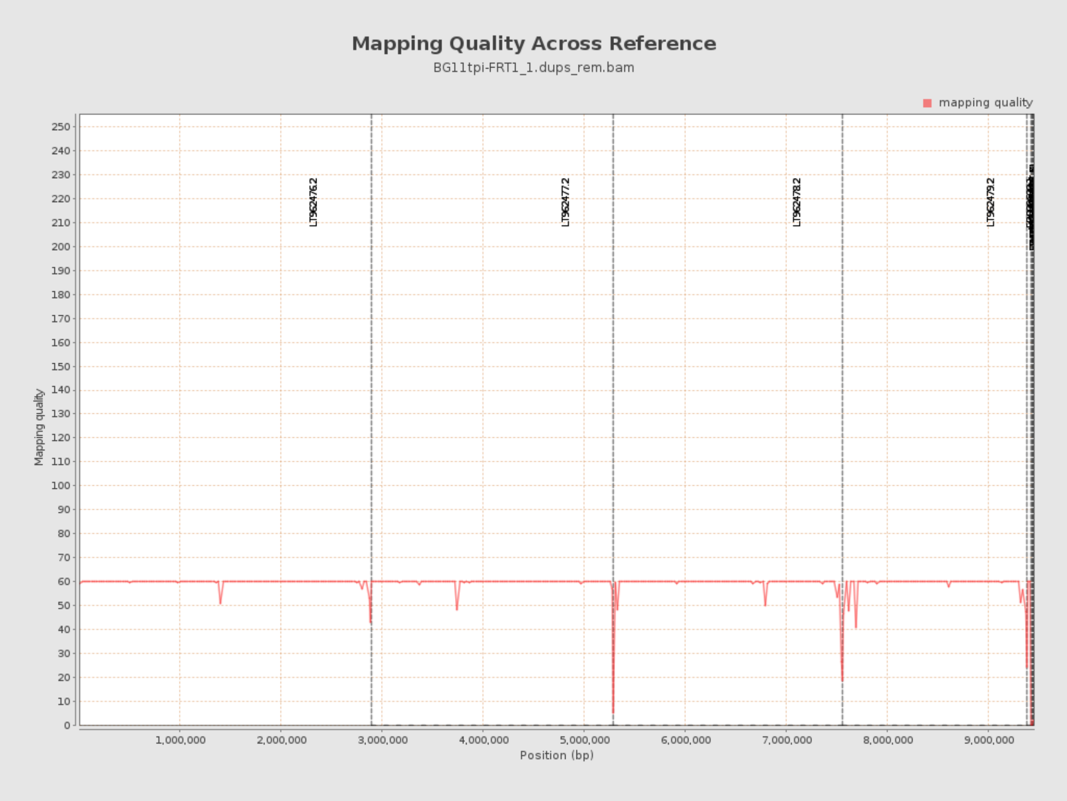

13. Results : Mapping Quality Histogram

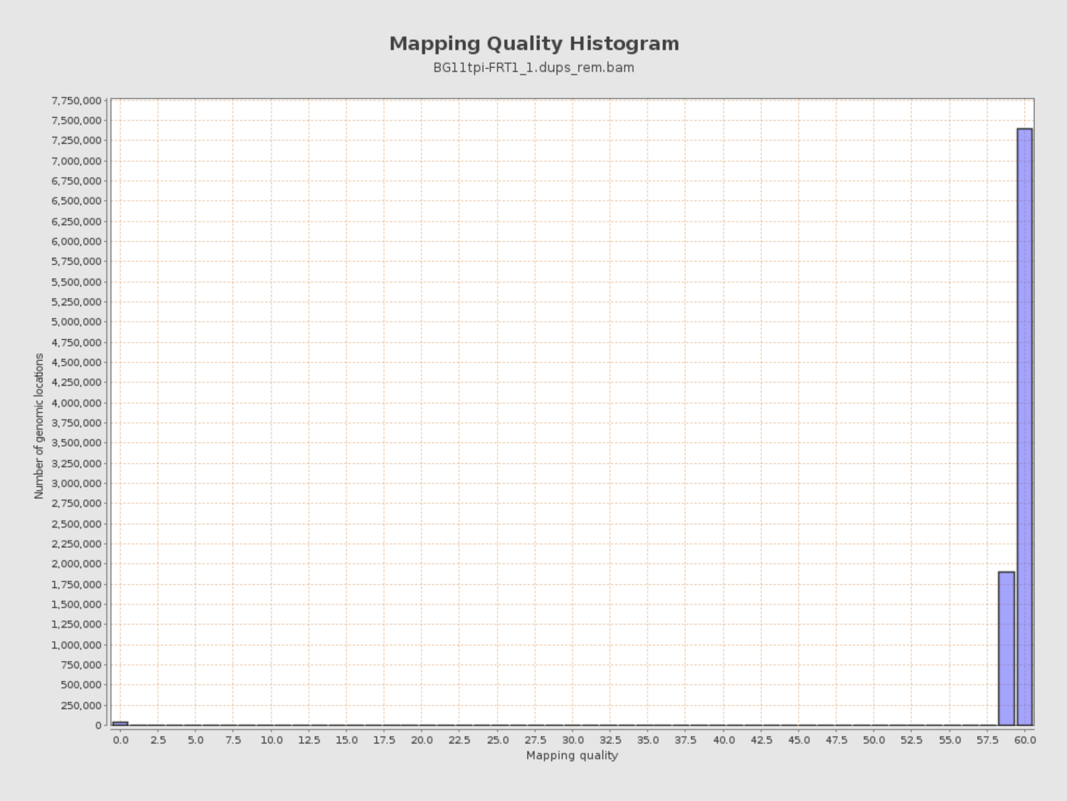

14. Results : Insert Size Across Reference

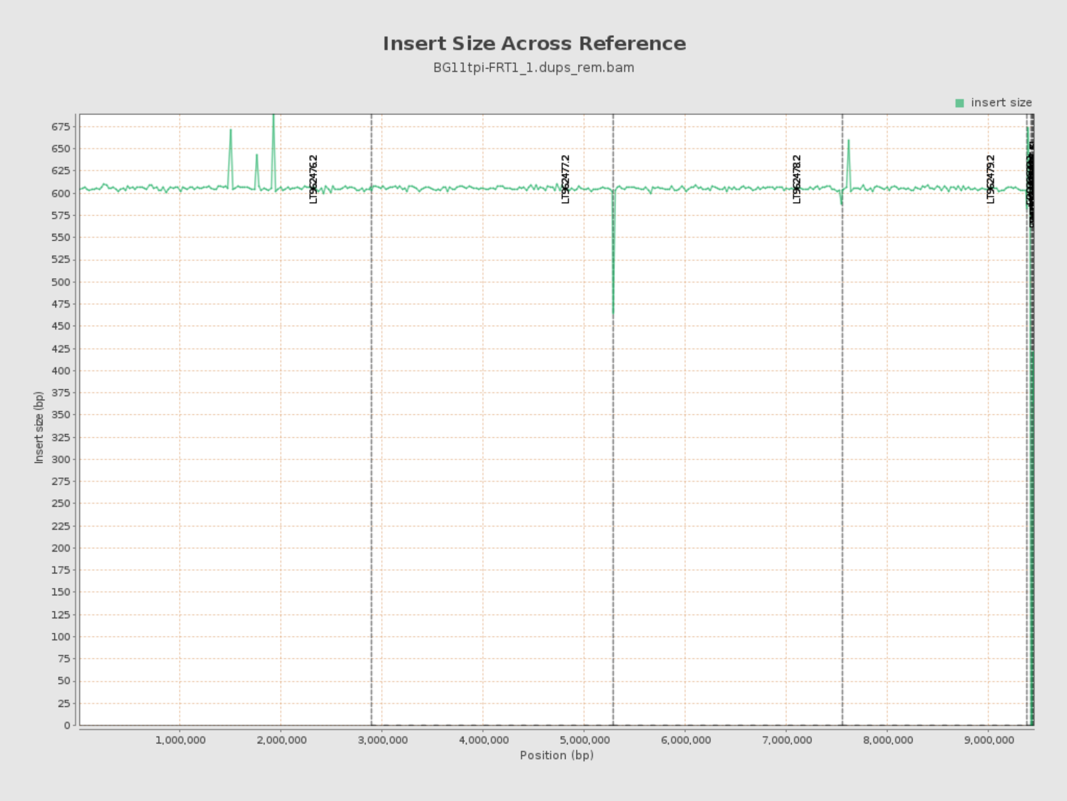

15. Results : Insert Size Histogram

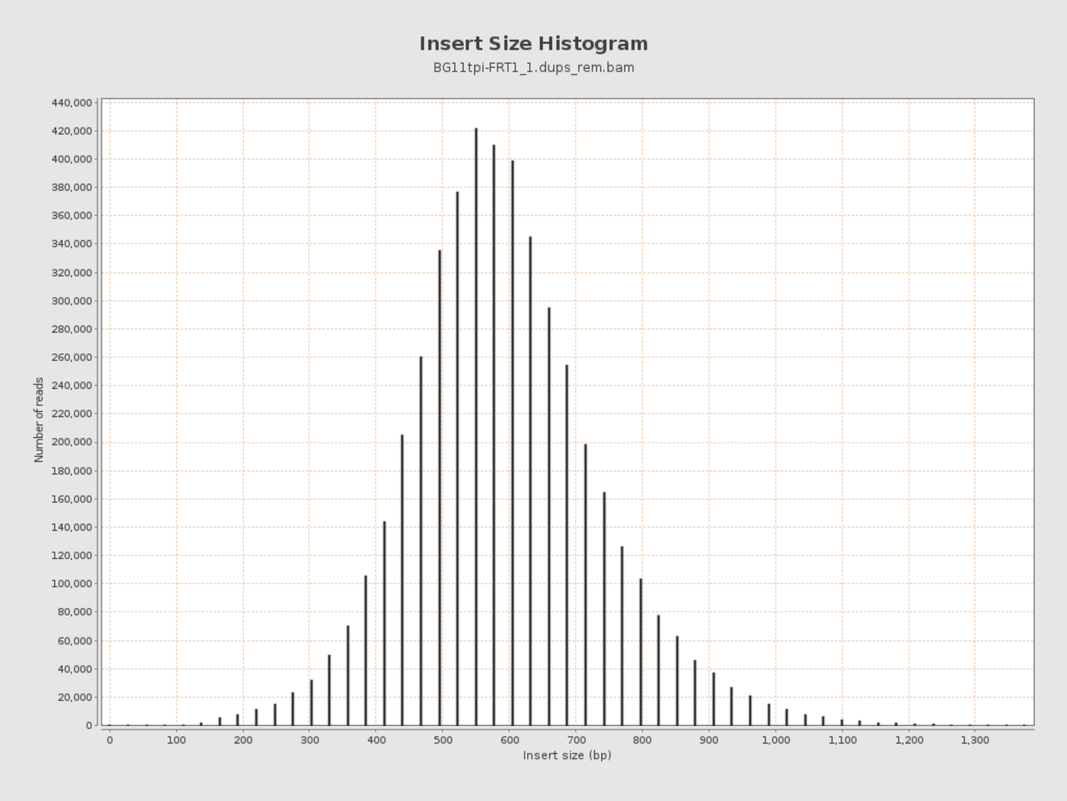

Supplement: Supplementary file 2 — Supplementary Material 2 [file 12934_2024_2368_MOESM2_ESM.zip › Supplementary_File_2/BSYBG11tpi1/BG11tpi-FRT1_1_qualimap.pdf]
